# Anti-Psoriatic Pharmacodynamic Material Basis of Dictamni Cortex Based on Transdermal Constituents Group

**DOI:** 10.3390/pharmaceutics17091195

**Published:** 2025-09-14

**Authors:** Zhaoyu Wang, Mengting Pi, Ziang Gao, Maobo Du, Liwei Gu, Shuzhi Liu, Shuo Shen

**Affiliations:** 1 State Key Laboratory for Quality Ensurance and Sustainable Use of Dao-di Herbs, Artemisinin Research Center, Beijing 100700, China; wzhaoyu6@gmail.com (Z.W.); pimengting61@gmail.com (M.P.); zianggao33@gmail.com (Z.G.); lwgu@icmm.ac.cn (L.G.); 2 Institute of Chinese Materia Medica, China Academy of Chinese Medical Sciences, Beijing 100700, China; mbdu@icmm.ac.cn (M.D.); liushuhi2004@sina.com (S.L.); 3 School of Traditional Chinese Materia Medica, Shenyang Pharmaceutical University, Shenyang 110016, China

**Keywords:** psoriasis, Dictamni Cortex, UPLC-Q-TOF-MS, transdermal administration, network pharmacology, HaCaT cell

## Abstract

**Background:** Psoriasis is a chronic inflammatory skin disorder for which topical medications are the preferred treatment option. However, current therapies are limited by adverse reactions, drug resistance, and economic burdens. Dictamni Cortex (DC; the root bark of *Dictamnus dasycarpus* Turcz.) has a long history in the treatment of psoriasis, with its transdermal bioactive constituents serving as the pharmacodynamic foundation for topical anti-psoriatic therapy. **Methods:** Building on the separation of DC’s chemical constituents, this study integrated ultra-performance liquid chromatography quadrupole time-of-flight mass spectrometry (UPLC-Q-TOF-MS) and network pharmacology, along with activity verification, to investigate the anti-psoriatic active components among the transdermal constituents of DC. **Results:** Forty-one chemical constituents were characterized in DC, including 26 transdermally permeable compounds, predominantly alkaloids and limonoids. Network pharmacological analysis revealed core targets, including MMP9 and TLR4, as well as multiple pathways related to inflammatory and immune responses. Molecular docking studies identified dictamnine, jangomolide, rutaevin, and other key transdermal constituents that exhibited high binding affinity to core targets. In vitro validation showed that these compounds significantly suppressed cellular proliferation (*p* < 0.05) and downregulated Ki67 mRNA expression (*p* < 0.05) in the psoriasis-like HaCaT cell model. Concurrently, they significantly reduced secretion of key pro-inflammatory cytokines, including IL-17A, IL-22, IL-1β, IL-6, and IL-8 (*p* < 0.05). Comprehensive comparative analyses confirmed that dictamnine exhibited ideal anti-psoriatic efficacy. **Conclusions:** These results provide a pharmacological substance basis for the development of external preparations of DC for treating psoriasis and provide novel research concepts for investigating the pharmacodynamic material basis of Traditional Chinese Medicine topical drugs.

## 1. Introduction

Psoriasis, a chronic inflammatory skin disorder with recurring outbreaks, impacts approximately 2–3% of the global population and shows a consistent increase in incidence [1]. The etiology and pathogenesis of the disease are complex and multifactorial, involving genetic, immunological, and environmental elements, and have not been fully elucidated, making a complete cure presently unattainable [2,3]. Moreover, accumulating evidence indicates that psoriasis is associated with comorbidities such as cardiovascular diseases [4], metabolic syndrome [5], and psychiatric disorders [6,7], which complicates treatment selection.

Psoriasis manifests with excessive keratinocyte hyperproliferation, infiltration of immune cells, and exaggerated inflammatory responses. In clinical settings, the disorder is categorized into subtypes: vulgaris, pustular, erythrodermic, and arthritic, with vulgaris being the most common. Although this subtype is not typically life-threatening, patients suffer from persistent skin lesions and recurrent flares. These symptoms severely reduce quality of life and create a substantial physiological and psychological burden for both patients and their families [8,9].

Current therapeutic strategies for psoriasis encompass topical agents, phototherapy, systemic drugs, and biologics that modulate immune pathways, aiming to mitigate symptoms and enhance patient well-being. Nevertheless, their clinical utility is constrained by adverse effects, drug resistance, long-term toxicity risks, and high costs, posing significant challenges in disease management [10,11].

Traditional Chinese medicine (TCM) has a long history and distinctive features in the management of psoriasis, with its earliest documented description preserved in the Zhu Bing Yuan Hou Lun (Treatise on the Causes and Symptoms of Diseases), a medical text originating from the Sui Dynasty (581–618 CE). Through millennia of clinical practice, TCM has accumulated extensive experience and has demonstrated advantages such as harmonizing therapeutic actions, favorable safety profiles, and evidence-based efficacy in psoriasis treatment. Clinical evidence has indicated that the accumulation of dampness-heat toxin in the skin induces qi-blood stasis, resulting in epidermal thickening and scale adhesion, which constitutes the core pathophysiology of psoriasis. Consequently, treatment strategies focus on heat-clearing and blood-cooling, blood-activating and stasis-resolving, as well as wind-dampness dispelling. TCM herbs exerting these therapeutic actions have demonstrated clinical efficacy in ameliorating psoriatic lesions [12,13,14].

Dictamni Cortex (DC), the dried root bark of *Dictamnus dasycarpus* Turcz. (Rutaceae), exhibits heat-clearing, dampness-drying, wind-dispelling, and detoxifying properties [15]. It was first documented in Shennong Bencao Jing (The Divine Farmer’s Materia Medica). Its primary active constituents encompass alkaloids, limonoids, flavonoids, and volatile oils, which provide a substantial pharmacological foundation. Contemporary pharmacological research has validated DC’s multifunctional bioactivities, including anti-inflammatory, antimicrobial, antiallergic, anticancer, pesticidal, and antioxidative properties [16,17]. DC is primarily used in dermatological practice and has a long history in psoriasis treatment, with documentation in both ancient medical texts and modern research. Yaoxing Lun (Drug Property Theory) and Bencao Yuanshi (Original Herbal Studies) state that it “treats diverse heat-toxins, pathogenic winds, wind-type sores, scabies, and erythematous erosions.” Additionally, Bencao Gangmu (Compendium of Materia Medica) documents that topical application of DC processed with wine is used to treat scabies. DC also plays an important role in the topical management of psoriasis in modern clinical practice [18,19]. A variety of topical lotions used by Dr Wang Yuxi for the treatment of psoriasis contain DC, which highlights its important role in the clinical management of this condition [20]. Basic research indicates that topical application of DC methanolic extract attenuates psoriasiform scaling and alleviates skin inflammation in psoriasis model mice [21]. Moreover, DC aqueous extract markedly ameliorates the inflammatory response in psoriatic rats via targeting Akt1, TP53, and Caspase-3 [22]. Dictamnine, the main constituent of DC, directly inhibits the potential target interleukin-23 (IL-23) of psoriasis [23]. These findings highlight that DC exhibits substantial therapeutic potential in psoriasis management and holds promise as a valuable candidate for the development of novel anti-psoriatic therapeutics.

The transdermally active components of DC are pivotal for its local therapeutic effects. However, due to the complex composition of TCM, the specific constituents responsible for its efficacy in transdermal psoriasis treatment have not yet been elucidated. Therefore, to elucidate the pharmacodynamic material basis underlying its anti-psoriatic efficacy, this study employed ultra-performance liquid chromatography-quadrupole time-of-flight mass spectrometry (UPLC-Q-TOF-MS) technology to accurately identify the transdermal constituents of DC, based on the systematic isolation of its chemical constituents. The potential therapeutic targets and signaling pathways of DC’s permeable constituents in psoriasis treatment were then systematically analyzed using network pharmacology approaches. Furthermore, molecular docking simulations were employed to characterize the interactions between chemical constituents and core targets. Subsequently, the anti-psoriasis active constituents were screened by anti-proliferation and anti-inflammatory experiments of the psoriasis-like human immortalized keratinocyte (HaCaT) cell model. This study provides novel research concepts for investigating the pharmacodynamic material basis of topical drugs, offering new perspectives for developing anti-psoriatic TCM topical preparations.

## 2. Materials and Methods

### 2.1. Materials and Reagents

*Dictamnus dasycarpus* Turcz. Root bark (Lot #2405001, Liaoning Province) was supplied by Xinjingyuan Pharmaceutical Co., Ltd. (Baoding, China). Dr. Shi Yuhua (Associate Researcher, National Resource Center for Chinese Materia Medica, China Academy of Chinese Medical Sciences) confirmed its botanical identity.

Male Kunming mice (SPF grade, 16–18 g) were obtained from Vital River Laboratory Animal Technology (Beijing, China; License No. SYXK [Jing] 2023-0077).

HaCaT cells and Minimum Essential Medium (MEM) were sourced from Saiku Biotechnology (Guangzhou, China). Recombinant human TNF-α was acquired from PeproTech (Cranbury, NJ, USA). Fetal bovine serum (FBS) and Cell Counting Kit-8 (CCK-8) came from Dalian Meilun Biotechnology (Dalian, China), while Penicillin-streptomycin (PS) solution and 0.25% trypsin (1×) solution were obtained from Gibco (Thermo Fisher Scientific, Waltham, MA, USA). RT SuperMix was purchased from Exongen (Chengdu, China). Enzyme-linked immunosorbent assay (ELISA) kits for Human interleukin-17 (IL-17), interleukin-22 (IL-22), interleukin-1β (IL-1β), interleukin-6 (IL-6), and interleukin-8 (IL-8) were supplied by Baiaosike Biotechnology (Beijing, China).

γ-Fagarine was provided by our laboratory, with rutaevin from Pusi Biotechnology (Chengdu, China) and calcipotriol from Yuanye Biotechnology (Shanghai, China). Additional reagents were purchased from Titan Technology (Shanghai, China). Preparative-grade reagents were used for semi-preparative chromatography, with chromatographic-grade materials for UPLC-Q-TOF-MS analyses.

### 2.2. Extraction, Separation, and Structural Identification of Chemical Constituents from DC

DC pieces (1.0 kg) were extracted three times with 70% ethanol (8.0 L, 8.0 L, and 6.0 L, respectively). Following extraction and filtration, the filtrate was rotary evaporated at 60 °C and further concentrated under reduced pressure in a 60 °C water bath until no ethanol odor remained, yielding 369 g of DC crude extract. The compounds were isolated by semi-preparative HPLC (Huide Yi Technology Co., Ltd., Beijing, China) using a binary gradient system of acetonitrile and water. Structural characterization was performed by ^1^H and ^13^C NMR spectroscopy (600 MHz; Bruker BioSpin GmbH, Fällanden, Switzerland), and the spectra were processed and analyzed using MestReNova (version 15.0.0) for structural determination.

### 2.3. Preparation of Test Solution

Precisely 0.15 g of the 70% ethanolic DC extract was transferred to a sealed conical flask, followed by the addition of 25 mL 70% ethanol and recording of the total mass. Ultrasonic-assisted extraction was performed for 30 min (300 W, 40 kHz) using a bath sonicator, after which the sample was allowed to equilibrate to ambient temperature. The flask was reweighed, and solvent loss was compensated for by adding fresh 70% ethanol. After homogenization, the sample was filtered (0.22 μm) to prepare the DC test solution.

The modified Franz diffusion cell technique was employed: intact abdominal skin from depilated mice was excised and securely mounted between the diffusion cell and the receiving cell. 0.50 g of DC 70% ethanol extract was added to the diffusion cell, with a diffusion area of 3.14 cm^2^. The receiving medium consisted of 95% ethanol-water (3:7, *v*/*v*). The transdermal permeation study was conducted using a TK-24BL diffusion system (Shanghai Kaikai Technology Co., Ltd., Shanghai, China) under precisely controlled conditions: temperature maintained at 32.0 ± 0.2 °C with continuous magnetic agitation (320 rpm). After 24 h of permeation, the receptor phase was carefully withdrawn, followed by sterile filtration (0.22 μm) to obtain the final DC permeation sample.

### 2.4. Identification of Transdermal Constituents of DC

UPLC-Q-TOF-MS (Waters Corporation, Milford, MA, USA) was employed for the qualitative identification of chemical constituents in DC ethanol extract and transdermal receiving solutions.

#### 2.4.1. Chromatographic Conditions

Chromatographic separation was achieved using an ACQUITY UPLC BEH C18 column (2.1 × 100 mm, 1.7 μm; Waters Corporation, Milford, MA, USA) with a mobile phase of (A) acetonitrile and (B) 0.1% aqueous formic acid. The following gradient program was applied: 5–100% A (0–20 min), maintaining 100% A (20–25 min). Chromatographic analysis was performed under the following conditions: a constant flow rate of 0.3 mL/min, with the column thermostat set to 35 °C and the autosampler maintained at 20 °C. Identical injection volumes (4 μL) were used for analysis of both DC crude extract and transdermal permeation samples, employing UV detection at 245 nm.

#### 2.4.2. Mass Spectrometric Analysis Parameters

The mass spectrometric analysis was conducted with an ESI interface operating in dual-polarity mode. Optimized ionization parameters included: desolvation gas (800 L/h, 450 °C), cone gas (50 L/h), capillary voltages (±3.0 kV for positive/−2.5 kV for negative mode), and source conditions (40 V cone voltage, 120 °C). Full-scan mass spectra were recorded at a 0.2 Hz acquisition rate across *m*/*z* 50–1500.

#### 2.4.3. Data Processing and Compound Identification

MassLynx (version 4.2) was employed for processing and interpreting the raw mass spectrometry data. In the base peak intensity (BPI) chromatograms, target compounds were identified by matching retention time (RT), accurate molecular weight (error < 5 ppm, theoretical value calculated using ChemDraw Professional 22.2.0), and characteristic fragment ions (MS/MS spectra). These data were compared with the chemical constituents isolated in Section 2.2 of this study and with reported characteristic compounds of DC in the literature to achieve compound identification.

### 2.5. Network Pharmacology Investigation

#### 2.5.1. Acquisition of Potential Targets for DC’s Transdermal Constituents

Following UPLC-Q-TOF-MS characterization, compounds demonstrating transdermal permeability were advanced to target prediction analysis. Initially, the compounds’ standard SMILES were obtained from PubChem (https://pubchem.ncbi.nlm.nih.gov/, accessed on 11 June 2025) [24]. Subsequent target profiling was performed using the SwissTargetPrediction platform (species: Homo sapiens; http://www.swisstargetprediction.ch/, accessed on 11 June 2025) [25], where all predictions with probability scores >0 were considered. The resulting targets were consolidated and duplicates removed, generating the final candidate target pool for DC’s transdermal constituents [26].

#### 2.5.2. “Transdermal Constituent-Target” Network Construction and Analysis

The DC transdermal component-target network was constructed in Cytoscape (version 3.10.2) [27], with topological analysis conducted using Network Analyzer. Node significance was assessed via degree centrality [28].

#### 2.5.3. Acquisition of Psoriasis Disease Targets

Potential therapeutic targets for psoriasis were systematically curated from established biomedical databases: DrugBank (https://go.drugbank.com/), GeneCards (https://www.genecards.org/), OMIM (https://www.omim.org/), and the Therapeutic Target Database (TTD, http://db.idrblab.net/ttd/). All databases were accessed on June 12, 2025 [29,30,31,32]. The retrieved targets were integrated and deduplicated to generate a psoriasis disease target set. In GeneCards, targets with a relevance score above the mean value were selected to ensure target relevance [33,34].

#### 2.5.4. Venn Diagram Construction for Constituent-Disease Intersection Targets

Common targets shared between DC’s transdermal components and psoriasis-related targets were determined through Venn diagram analysis using Venny 2.1.0 (https://bioinfogp.cnb.csic.es/tools/venny/, accessed on 15 June 2025) [34,35].

#### 2.5.5. PPI Network Establishment and Core Target Identification

The common targets were analyzed using the STRING platform (https://string-db.org/, accessed on 16 June 2025) to generate a Protein–Protein Interaction (PPI) network [36]. Homo sapiens were specified with interaction confidence set at >0.400. After eliminating isolated nodes and non-interacting partners, the network was visualized and analyzed in Cytoscape (version 3.10.2). Topological assessment employed median values of degree, closeness centrality, and betweenness centrality as selection criteria. Nodes satisfying all three cutoff criteria simultaneously were identified as core targets [37,38].

#### 2.5.6. Functional Annotation and Pathway Enrichment Analysis

Core targets were functionally annotated via Metascape (https://metascape.org/gp/index.html, accessed 17 June 2025) using *p* < 0.01 as the significance cutoff [39]. The Gene Ontology (GO) analysis systematically examined biological processes (BP), cellular components (CC), and molecular functions (MF), while Kyoto Encyclopedia of Genes and Genomes (KEGG) pathway mapping identified key signaling pathways implicated in psoriasis pathogenesis [40,41]. The Bioinformatics online resource (http://www.bioinformatics.com.cn/, accessed on 18 June 2025) was employed to visualize the top 10–20 most significantly enriched terms based on *p* values [42].

#### 2.5.7. Construction and Analysis of Multidimensional Interaction Network

A comprehensive network integrating percutaneous components, pivotal targets, and associated signaling pathways was established using Cytoscape (version 3.10.2), visualizing the multidimensional relationships among these elements. This was performed to further explore the molecular mechanisms of transdermal administration of DC in the treatment of psoriasis [43].

#### 2.5.8. Molecular Docking

Target protein structures were acquired from RCSB PDB (https://www.rcsb.org/, accessed 20 June 2025) [44]. 2D molecular structures were retrieved from PubChem, followed by energy optimization in Chem3D, while PyMOL (version 3.1.0) was used for protein preprocessing, including dehydration and ligand removal. Default parameters were applied for all subsequent steps. Protein and ligand preparations were conducted with AutoDockTools (version 1.5.7), followed by active site-centered grid generation. Molecular docking was executed using AutoDock Vina [45], with subsequent visualization of binding poses in PyMOL (version 3.1.0).

### 2.6. In Vitro Validation

#### 2.6.1. TNF-α-Stimulated HaCaT Cell Model Construction

HaCaT cells, an immortalized human keratinocyte line, were maintained in complete growth medium (MEM with 10% FBS and 1% PS) under standard culture conditions (37 °C, 5% CO_2_, humidified atmosphere). Upon reaching 80–90% confluence, adherent cells were detached using 0.25% trypsin for subculture. For proliferation assessment, cells (1 × 10^4^/well) were cultured overnight in 96-well plates to ensure adhesion. Following attachment, the culture medium was exchanged with TNF-α-containing MEM (10–50 ng/mL final concentration) for 24 h treatment. Cell viability quantification was performed using the CCK-8 method: after adding 10 μL CCK-8 solution per well, plates were incubated for 30–60 min before measuring optical density at 450 nm using a microplate reader. The viability percentage was calculated using the following formula:

Cell viability (%) = [(OD_450_(experimental) − OD_450_(blank))/(OD_450_(control) − OD_450_(blank))] × 100%

HaCaT keratinocytes were plated in 6-well culture dishes (3 × 10^5^ cells/well) and allowed to adhere. The culture medium was then replaced with serum-free MEM containing TNF-α (10–50 ng/mL) for 24 h stimulation. For cell harvesting, monolayers were rinsed with phosphate-buffered saline (PBS) and dissociated using 0.25% trypsin-EDTA (1 mL/well, 37 °C, 5–10 min), with enzymatic activity subsequently neutralized by adding 2 volumes of complete medium. Cell counts were determined in triplicate measurements using an automated cell counting system (Cellometer Mini, Nexcelom Bioscience, Lawrence, MA, USA) [46].

HaCaT keratinocytes were plated in 6-well culture dishes (3 × 10^5^ cells/well) and exposed to TNF-α (30 ng/mL) or vehicle control for 24 h. Culture supernatants were subsequently harvested for quantification of IL-1β and IL-6 secretion using manufacturer-specified protocols. Additional experimental conditions employed TNF-α at concentrations up to 30 ng/mL as the highest treatment dose.

#### 2.6.2. Impact of Investigational Agents on Keratinocyte Viability

For experimental procedures, 96-well plates were inoculated with HaCaT cells at a density of 1 × 10^4^ cells per well. Following a 24 h incubation period, complete cellular adhesion was achieved prior to subsequent treatment. Cells were exposed to serially diluted concentrations of the test compounds. An untreated control group (cells only) and a cell-free blank group were included. After 24 h of treatment, the CCK-8 assay was performed as described in Section 2.6.1. OD_450_ measurements were obtained for viability calculations. Cytotoxic effects of different compound concentrations were evaluated, and appropriate concentrations were selected for subsequent experiments based on the viability data.

#### 2.6.3. Impact of Investigational Agents on TNF-α-Mediated Keratinocyte Growth

For experimental procedures, 96-well plates were inoculated with HaCaT cells at a density of 1 × 10^4^ cells per well. Following a 24 h incubation period, complete cellular adhesion was achieved prior to subsequent treatment. Experimental groups included: (1) untreated control (cells only); (2) TNF-α model group (30 ng/mL TNF-α alone); (3) test compound groups (30 ng/mL TNF-α + test compounds at final concentrations of 12.5, 25, and 50 μM); and (4) cell-free blank group. CCK-8 viability assessment was performed after 24 h of co-treatment. The inhibitory effects of test compounds on TNF-α-induced cell proliferation were evaluated based on cell viability data.

#### 2.6.4. Extraction of RNA and Reverse Transcription Quantitative Polymerase Chain Reaction (RT-qPCR)

Total RNA isolation was performed using TRIzol (Aidlab biotechnologies Co., Ltd., Beijing, China) reagent following the manufacturer’s protocol. For cDNA synthesis, 1 μg of extracted RNA was reverse transcribed in a 20 μL reaction volume containing: 1 μg RNA template, 4 μL of 5× Reaction Mix, 3 μL Supreme Enzyme Mix, and DEPC-treated water to volume. The reverse transcription protocol comprised: 25 °C (10 min), 55 °C (15 min), and 85 °C (5 min), with resulting cDNA stored at −20 °C. Quantitative real-time PCR analysis was conducted using the Langji Real-Time PCR System (Hangzhou, China) [47]. The thermal cycling protocol included: initial denaturation at 95 °C for 5 min; 40 cycles of 95 °C for 10 s, 58 °C for 20 s, and 72 °C for 20 s; followed by melting curve analysis (95 °C for 15 s, 60 °C for 60 s, and 95 °C for 15 s). mRNA expression levels were determined using the comparative threshold cycle (Ct) method, with relative expression calculated using the 2^−ΔΔCt^ method and β-actin as the housekeeping gene. The qPCR primer sequences are listed in Table 1.

#### 2.6.5. ELISA Detection of Inflammatory Markers

According to the experimental grouping described in Section 2.6.3, HaCaT keratinocytes were plated in 6-well culture dishes (1 × 10^6^ cells/well). Conditioned media were harvested from TNF-α-activated cultures following compound treatment for subsequent cytokine quantification. Concentrations of interleukin IL-17A, IL-22, IL-1β, IL-6, and IL-8 were determined using commercial ELISA kits following the supplier’s protocols.

#### 2.6.6. Data Analysis

Statistical analyses were conducted with GraphPad Prism (version 8.0), with data expressed as mean ± SD (*n* = 3). Intergroup differences were analyzed by one-way ANOVA, with statistical significance defined as *p* < 0.05.

## 3. Results and Discussion

### 3.1. Structure Identification of Extracted Constituents

**Figure 1:**
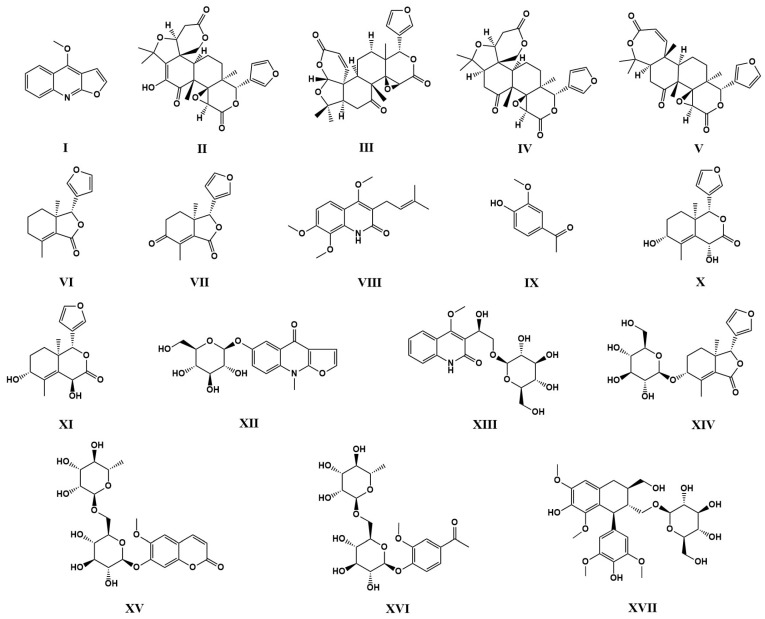
Structures of compounds **I**–**XVII** from the 70% ethanol extract of DC.

The 70% ethanol extract of DC was fractionated using macroporous resin to obtain fractions with different polarities. Further separation and purification by semi-preparative HPLC led to the isolation of seventeen compounds. Their structures were identified by NMR and ESI-MS. The seventeen compounds were as dictamnine (I) [48,49], evodol (II) [50], jangomolide (III) [51], limonin (IV) [48], obacunone (V) [52], fraxinellone (VI) [48], fraxinellonone (VII) [52,53], preskimmianine (VIII) [50,54], acetovanillone (IX) [55], isodictamdiol C (X) [56], dictamdiol B (XI) [57], dictamalkoside A (XII) [58], 3-[1β-hydroxy-2-(β-D-glucopyranosyloxy)-ethyl)-4-methoxy2(1*H*)-quinolinone (XIII) [59], 9α-hydroxyfraxinellone-9-O-β-D-glucoside (XIV) [57], haploperoside A (XV) [60,61], 4-hydroxy-3-methoxy-acetophenone-4-O-α-L-rhamnopyranosyl-(1→6)-β-D-glucopyranoside (XVI) [62], (+)-lyoniresinol-3α-O-β-D-glucopyranoside (XVII) [63]. Structural details are provided in Figure 1, and nuclear magnetic resonance spectra are provided in Supplementary Figures S1–S34.

Compound **I**: dictamnine (4-methoxyfuro[2,3-b]quinoline), white needle crystals, mp 126~128 °C. ESI-MS *m*/*z*: [M+H]^+^ 200.0713 (calcd for C_12_H_9_NO_2_, 200.0712). ^1^H NMR (600 MHz, DMSO-*d*_6_), *δ*: 8.15 (dd, *J* = 8.4, 1.5 Hz, 1H), 8.01 (d, *J* = 2.8 Hz, 1H), 7.88 (dd, *J* = 8.5, 0.7 Hz, 1H), 7.68 (ddd, *J* = 8.4, 6.7, 1.5 Hz, 1H), 7.44 (ddd, *J* = 8.3, 6.7, 1.2 Hz, 1H), 7.41 (d, *J* = 2.8 Hz, 1H), 4.40 (s, 3H). ^13^C NMR (150 MHz, DMSO-*d*_6_), *δ*: 163.4, 156.2, 144.9, 144.3, 129.4, 127.3, 123.6, 122.1, 118.0, 105.4, 103.3, 59.3. The above data are consistent with those reported in References [48,49], thus the compound is identified as dictamnine.

Compound **II**: evodol ((1*R*,2*R*,7*S*,13*R*,14*R*,16*S*,19*S*,20*S*)-19-(furan-3-yl)-11-hydrox-9,9,13,20-tetramethyl-4,8,15,18-tetraoxahexacyclo[11.9.0.0^2,7^.0^2,10^.0^14,16^.0^14,20^]docos-10-ene-5,12,17-trione), white needle crystals, mp 279~281 °C, ESI-MS *m*/*z*: [M−H]^−^ 483.166 (calcd for C_26_H_28_O_9_, 483.1655). ^1^H NMR (600 MHz, CDCl_3_), *δ*: 7.41 (s, 1H), 7.40 (t, *J* = 1.7 Hz, 1H), 6.33 (s, 1H), 6.30 (s, 1H), 5.44 (s, 1H), 4.60–4.68 (m, 2H), 4.12 (s, 1H), 4.07 (s, 1H), 2.97 (dd, *J* = 18.1, 2.5 Hz, 1H), 2.84 (dd, *J* = 18.0, 4.4 Hz, 1H), 2.67 (dd, *J* = 13.2, 2.1 Hz, 1H), 1.80–1.97 (m, 2H), 1.59–1.72 (m, 2H), 1.55 (s, 3H), 1.50 (s, 3H), 1.16 (s, 3H), 1.05 (s, 3H). ^13^C NMR (150 MHz, CDCl_3_), *δ*: 195.3, 169.3, 166.6, 143.5, 141.2, 140.2, 139.6, 119.9, 109.8, 81.9, 79.3, 77.8, 68.7, 65.4, 52.2, 48.5, 47.0, 46.5, 37.5, 34.9, 31.8, 25.8, 25.3, 20.8, 20.6, 18.2. The above data are consistent with those reported in Reference [50], thus the compound is identified as evodol.

Compound **III**: jangomolide ((1*R*,2*S*,7*R*,10*R*,13*R*,14*R*,16*S*,19*S*,20*S*)-19-(furan-3-yl)-9,9,13,20-tetramethyl-6,8,15,18-tetraoxahexacyclo[11.9.0.0^2,7^.0^2,10^.0^14,16^.0^14,20^]docos-3-ene-5,12,17-trione), colorless crystals, mp 270~272 °C. ESI-MS *m*/*z*: [M+H]^+^ 469.1862 (calcd for C_26_H_28_O_8_, 469.1862). ^1^H NMR (600 MHz, CDCl_3_), *δ*: 7.38–7.43 (m, 2H), 6.52 (d, *J* = 10.0 Hz, 1H), 6.32 (s, 1H), 6.13 (d, *J* = 9.9 Hz, 1H), 6.06 (s, 1H), 5.53 (s, 1H), 3.99 (s, 1H), 2.69 (d, *J* = 6.2 Hz, 2H), 2.64–2.67 (m, 1H), 2.61 (t, *J* = 6.2 Hz, 1H), 1.75–1.79 (m, 2H), 1.49–1.53 (m, 2H), 1.36 (s, 3H), 1.28 (s, 6H), 1.16 (s, 3H). ^13^C NMR (150 MHz, CDCl_3_), *δ*: 209.0, 166.7, 160.4, 150.7, 143.4, 141.3, 120.0, 119.2, 109.8, 104.1, 86.5, 77.9, 67.4, 54.3, 53.4, 49.5, 49.4, 42.0, 38.6, 37.8, 31.8, 29.3, 25.0, 20.4, 19.3, 15.8. The above data are consistent with those reported in Reference [51], thus the compound is identified as jangomolide.

Compound **IV**: limonin ((1*R*,2*R*,7*S*,10*R*,13*R*,14*R*,16*S*,19*S*,20*S*)-19-(furan-3-yl)-9,9,13,20-tetramethyl-4,8,15,18-tetraoxahexacyclo[11.9.0.0^2,7^.0^2,10^.0^14,16^.0^14,20^]docosane-5,12,17-trione), white plate-like crystals, mp 289~291 °C. ESI-MS *m*/*z*: [M+H]^+^ 471.2020 (calcd for C_26_H_30_O_8_, 471.2019). ^1^H NMR (600 MHz, DMSO-*d*_6_), *δ*: 7.72 (s, 1H), 7.66 (t, *J* = 1.7 Hz, 1H), 6.51 (d, *J* = 1.0 Hz, 1H), 5.48 (s, 1H), 4.92 (d, *J* = 11.9 Hz, 1H), 4.48 (d, *J* = 13.0 Hz, 1H), 4.11 (d, *J* = 5.7 Hz, 2H), 3.12 (t, *J* = 15.3 Hz, 1H), 2.77 (d, *J* = 15.1 Hz, 1H), 2.62 (dd, *J* = 16.4, 4.1 Hz, 1H), 2.57 (dd, *J* = 12.5, 3.4 Hz, 1H), 2.46 (dd, *J* = 15.7, 3.4 Hz, 1H), 2.28 (dd, *J* = 14.9, 3.4 Hz, 1H), 1.79–1.86 (m, 1H), 1.69–1.75 (m, 2H), 1.22–1.26 (m, 1H), 1.19 (s, 3H), 1.11 (s, 3H), 1.02 (s, 3H), 1.00 (s, 3H). ^13^C NMR (150 MHz, DMSO-*d*_6_), *δ*: 208.1, 170.2, 167.3, 143.4, 141.7, 120.2, 110.2, 79.5, 78.4, 77.4, 66.7, 64.8, 58.0, 53.7, 50.3, 46.5, 45.3, 37.6, 36.2, 35.7, 29.8, 29.2, 21.4, 19.7, 17.5, 17.0. The above data are consistent with those reported in Reference [48], thus the compound is identified as limonin.

Compound **V**: obacunone ((1*R*,2*R*,4*S*,7*S*,8*S*,11*R*,12*R*,18*R*)-7-(furan-3-yl)-1,8,12,17,17-pentamethyl-3,6,16-trioxapentacyclo[9.9.0.0^2,4^.0^2,8^.0^12,18^]icos-13-ene-5,15,20-trione), colorless crystals, mp 229~231 °C. ESI-MS *m*/*z*: [M+H]^+^ 455.2070 (calcd for C_26_H_30_O_7_, 455.2070). ^1^H NMR (600 MHz, MeOD), *δ*: 7.54 (s, 1H), 7.50 (t, *J* = 1.8 Hz, 1H), 6.78 (d, *J* = 11.8 Hz, 1H), 6.46 (d, *J* = 1.1 Hz, 1H), 5.93 (d, *J* = 11.8 Hz, 1H), 5.54 (s, 1H), 3.69 (s, 1H), 3.14 (t, *J* = 14.1 Hz, 1H), 2.73 (dd, *J* = 14.1, 5.0 Hz, 1H), 2.29 (dd, *J* = 14.1, 5.0 Hz, 1H), 2.14–2.25 (m, 2H), 1.87–1.95 (m, 3H), 1.52 (s, 3H), 1.50 (s, 3H), 1.44 (s, 3H), 1.28 (s, 3H), 1.12 (s, 3H). ^13^C NMR (150 MHz, MeOD), *δ*: 209.7, 169.7, 169.4, 160.1, 144.4, 142.7, 122.7, 121.8, 111.0, 86.1, 79.7, 66.6, 58.2, 54.4, 54.3, 50.3, 44.6, 40.9, 38.7, 33.6, 32.2, 27.1, 21.4, 20.4, 17.3, 16.8. The above data are consistent with those reported in Reference [52], thus the compound is identified as obacunone.

Compound **VI**: fraxinellone ((3*R*,3*aR*)-3-(furan-3-yl)-3*a*,7-dimethyl-3,4,5,6-tetrahydr-o-2-benzofuran-1-one), white needle crystals, mp 114~116 °C. ESI-MS *m*/*z*: [M+H]^+^ 233.1176 (calcd for C_14_H_16_O_3_, 233.1178). ^1^H NMR (600 MHz, CDCl_3_), *δ*: 7.47 (s, 1H), 7.43 (t, *J* = 1.8 Hz, 1H), 6.34 (d, *J* = 1.1 Hz, 1H), 4.88 (s, 1H), 2.23–2.31 (m, 1H), 2.14–2.30 (m, 1H), 2.13 (s, 3H), 1.78–1.89 (m, 2H), 1.69–1.77 (m, 1H), 1.41–1.49 (m, 1H), 0.85 (s, 3H). ^13^C NMR (150 MHz, CDCl_3_), *δ*: 170.0, 148.7, 143.5, 139.9, 127.5, 120.8, 108.7, 83.5, 43.1, 32.2, 31.8, 20.5, 18.6, 18.4. The above data are consistent with those reported in Reference [48], thus the compound is identified as fraxinellone.

Compound **VII**: fraxinellonone ((3*R*,3*aR*)-3-(furan-3-yl)-3*a*,7-dimethyl-4,5-dihydro-3*H*-2-benzofuran-1,6-dione), colorless needle and plate-like crystals, mp 119~121 °C, ESI-MS *m*/*z*: [M+H]^+^ 247.0966 (calcd for C_14_H_14_O_4_, 247.0970). ^1^H NMR (600 MHz, DMSO-*d*_6_), *δ*: 7.78 (s, 1H), 7.76 (t, *J* = 1.7 Hz, 1H), 6.57 (d, *J* = 2.2 Hz, 1H), 5.35 (s, 1H), 2.66–2.75 (m, 1H), 2.43–2.49 (m, 1H), 2.18–2.27 (m, 1H), 2.02 (s, 3H), 1.89–1.96 (m, 1H), 1.01 (s, 3H). ^13^C NMR (150 MHz, DMSO-*d*_6_), *δ*: 198.3, 168.7, 145.1, 144.2, 140.6, 137.6, 119.4, 109.0, 81.8, 43.4, 32.6, 30.8, 18.5, 9.7. The above data are consistent with those reported in References [52,53], thus the compound is identified as fraxinellonone.

Compound **VIII**: preskimmianine (4,7,8-trimethoxy-3-(3-methylbut-2-enyl)-1*H*-qui-nolin-2-one), white plate-like crystals, mp 122~124 °C. ESI-MS *m*/*z*: [M+H]^+^ 304.1546 (calcd for C_17_H_21_NO_4_, 304.1549). ^1^H NMR (600 MHz, DMSO-*d*_6_), *δ*: 10.85 (s, 1H), 7.41 (d, *J* = 9.0 Hz, 1H), 7.00 (d, *J* = 9.0 Hz, 1H), 5.16 (t, *J* = 7.8 Hz, 1H), 3.89 (s, 3H), 3.84 (s, 3H), 3.77 (s, 3H), 3.19 (d, *J* = 7.0 Hz, 2H), 1.73 (s, 3H), 1.64 (s, 3H). ^13^C NMR (150 MHz, DMSO-*d*_6_), *δ*: 163.6, 160.8, 152.7, 133.9, 132.0, 131.1, 122.0, 119.6, 118.2, 111.0, 107.6, 61.5, 60.6, 56.1, 25.5, 22.8, 17.8. The above data are consistent with those reported in References [50,54], thus the compound is identified as preskimmianine.

Compound **IX**: acetovanillone (1-(4-hydroxy-3-methoxyphenyl)ethenone), light yellow oily substance. ESI-MS *m*/*z*: [M+H]^+^ 167.0708 (calcd for C_9_H_10_O_3_, 167.0708). ^1^H NMR (600 MHz, CD_3_OD), *δ*: 7.56 (dd, *J* = 8.3, 2.1 Hz, 1H), 7.52 (d, *J* = 2.1 Hz, 1H), 6.82 (d, *J* = 8.3 Hz, 1H), 3.89 (s, 3H), 2.53 (s, 3H). ^13^C NMR (150 MHz, CD_3_OD), *δ*: 199.4, 154.9, 149.4, 129.8, 125.5, 116.1, 111.8, 56.3, 26.1. The above data are consistent with those reported in Reference [55], thus the compound is identified as acetovanillone.

Compound **X**: isodictamdiol C ((1*R*,4*R*,6*R*,8*aR*)-1-(furan-3-yl)-4,6-dihydroxy-5,8*a*-dimethyl-1,4,6,7,8,8*a*-hexahydro-3*H*-isochromen-3-one), light yellow solid. ESI-MS *m*/*z*: [M+K]^+^ 301.1045(calcd for C_15_H_18_O_5_, 301.1052). ^1^H NMR (600 MHz, DMSO-*d*_6_), *δ*: 7.71 (s, 1H), 7.67 (t, *J* = 1.8 Hz, 1H), 6.50 (d, *J* = 1.8 Hz, 1H), 5.72 (d, *J* = 5.5 Hz, 1H), 5.03 (s, 1H), 4.85 (d, *J* = 5.2 Hz, 1H), 4.81 (d, *J* = 5.7 Hz, 1H), 3.75 (s, 1H), 1.80 (s, 3H), 1.54–1.62 (m, 2H), 1.23 (s, 1H), 0.88 (s, 3H), 0.80 (dt, *J* = 12.5, 3.3 Hz, 1H). ^13^C NMR (150 MHz, DMSO-*d*_6_), *δ*: 173.5, 143.3, 141.4, 135.0, 133.8, 120.9, 110.1, 79.8, 65.8, 64.9, 38.0, 26.9, 26.5, 17.1, 15.5. The above data are consistent with those reported in Reference [56], thus the compound is identified as isodictamdiol C.

Compound **XI**: dictamdiol B ((1*R*,4*S*,6*R*,8*aR*)-1-(furan-3-yl)-4,6-dihydroxy-5,8*a*-dim-ethyl-4,6,7,8-tetrahydro-1*H*-isochromen-3-one), yellowish oil. ESI-MS *m*/*z*: [M+K]^+^ 301.1051 (calcd for C_15_H_18_O_5_, 301.1052). ^1^H NMR (600 MHz, DMSO-*d*_6_), *δ*: 7.73 (s, 1H), 7.68 (t, *J* = 1.7 Hz, 1H), 6.52 (d, *J* = 1.8 Hz, 1H), 6.37 (d, *J* = 4.8 Hz, 1H), 5.44 (s, 1H), 4.82 (d, *J* = 7.0 Hz, 1H), 4.65 (d, *J* = 4.3 Hz, 1H), 3.85 (q, *J* = 7.6 Hz, 1H), 1.88–1.94 (m, 1H), 1.73 (s, 3H), 1.57–1.65 (m, 1H), 1.44 (td, *J* = 13.8, 3.3 Hz, 1H), 0.97 (dt, *J* = 12.5, 3.3 Hz, 1H), 0.94 (s, 3H). ^13^C NMR (150 MHz, DMSO-*d*_6_), *δ*: 170.8, 143.4, 141.5, 138.0, 133.6, 120.0, 110.1, 78.5, 68.4, 66.2, 38.7, 31.2, 27.8, 18.4, 14.1. The above data are consistent with those reported in Reference [57], thus the compound is identified as dictamdiol B.

Compound **XII**: dictamalkoside A (9-methyl-6-(((2*S*,3*R*,4*S*,5*S*,6*R*)-3,4,5-trihydroxy-6-(hydroxymethyl)tetrahydro-2*H*-pyran-2-yl)oxy)furo[2,3-*b*]quinolin-4(9*H*)-one), yellow powder. ESI-MS *m*/*z*: [M+H]^+^ 378.1190 (calcd for C_18_H_19_NO_8_, 378.1189). ^1^H NMR (600 MHz, DMSO-*d*_6_), *δ*: 7.89 (d, *J* = 2.9 Hz, 1H), 7.79 (d, *J* = 9.2 Hz, 1H), 7.75 (d, *J* = 2.2 Hz, 1H), 7.54 (dd, *J* = 9.2, 3.0 Hz, 1H), 7.01 (d, *J* = 2.2 Hz, 1H), 4.94 (d, *J* = 7.4 Hz, 1H), 3.92 (s, 3H), 3.66–3.71 (m, 1H), 3.51 (dt, *J* = 11.4, 5.4 Hz, 1H), 3.34–3.35 (m, 1H), 3.33 (s, 1H), 3.29 (s, 1H), 3.16–3.25 (m, 1H). ^13^C NMR (150 MHz, DMSO-*d*_6_), *δ*: 171.1, 155.7, 153.0, 139.2, 133.5, 125.6 122.3, 117.1, 111.5, 107.2, 104.9, 101.4, 77.1, 76.5, 73.3, 69.6, 60.6, 31.6. The above data are consistent with those reported in Reference [58], thus the compound is identified as dictamalkoside A.

Compound **XIII**: 3-[1β-hydroxy-2-(β-D-glucopyranosyloxy)-ethyl)-4-methoxy2(1*H*)-Quinolinone (3-((*R*)-1-hydroxy-2-(((2*R*,3*R*,4*S*,5*S*,6*R*)-3,4,5-trihydroxy-6-(hydroxymethyl)-tetrahydro-2*H*-pyran-2-yl)oxy)ethyl)-4-methoxyquinolin-2(1*H*)-one), Yellowish gum. ESI-MS *m*/*z*: [M+H]^+^ 398.1450 (calcd for C_18_H_23_NO_9_, 398.1451). ^1^H NMR (600 MHz, DMSO-*d*_6_), *δ*: 7.74 (dd, *J* = 8.1, 1.4 Hz, 1H), 7.53 (ddd, *J* = 8.4, 7.1, 1.4 Hz, 1H), 7.35 (d, *J* = 8.6 Hz, 1H), 7.21–7.25 (m, 1H), 5.10 (td, *J* = 7.1, 4.6 Hz, 1H), 4.24 (d, *J* = 7.8 Hz, 1H), 3.92–3.99 (m, 5H), 3.63 (dq, *J* = 11.8, 2.1 Hz, 1H), 3.41 (dt, *J* = 11.4, 5.5 Hz, 2H), 3.12–3.17 (m, 1H), 3.09 (ddd, *J* = 9.9, 5.9, 2.2 Hz, 1H), 3.02 (td, *J* = 9.2, 3.7 Hz, 1H), 2.96 (td, *J* = 8.3, 3.1 Hz, 1H). ^13^C NMR (150 MHz, DMSO-*d*_6_), *δ*: 163.4, 162.4, 138.3, 130.8, 123.1, 122.0, 121.5, 116.0, 115.5, 103.5, 76.9, 76.6, 73.6, 72.4, 70.0, 65.9, 63.0, 61.1. The above data are consistent with those reported in Reference [59], thus the compound is identified as 3-[1β-hydroxy-2-(β-D-glucopyranosyloxy)-ethyl)-4-methoxy2(1*H*)-quinolinone.

Compound **XIV**: 9α-hydroxyfraxinellone-9-O-β-D-glucoside ((3*R*,3*aR*,6*R*)-3-(3-Fura-nyl)-6-(β-D-glucopyranosyloxy)-3*a*,4,5,6-tetrahydro-3*a*,7-dimethyl-1(3*H*)-isobenzofuranone), yellowish oil. ESI-MS *m*/*z*: [M+H]^+^ 411.1655 (calcd for C_20_H_26_O_9_, 411.1655). ^1^H NMR (600 MHz, DMSO-*d*_6_), *δ*: 7.72 (d, *J* = 2.1 Hz, 2H), 6.52 (s, 1H), 5.01 (s, 1H), 4.39 (d, *J* = 7.8 Hz, 1H), 4.01 (d, *J* = 4.4 Hz, 1H), 3.68 (d, *J* = 11.6 Hz, 1H), 3.40–3.48 (m, 1H), 3.12–3.18 (m, 2H), 3.04 (t, *J* = 9.3 Hz, 1H), 2.94–2.99 (m, 1H), 2.27 (dq, *J* = 14.6, 2.9 Hz, 1H), 2.17 (s, 3H), 1.80 (tt, *J* = 14.5, 4.0 Hz, 1H), 1.63 (td, J = 13.4, 3.3 Hz, 1H), 1.49 (dt, *J* = 12.5, 3.5 Hz, 1H), 0.76 (s, 3H). ^13^C NMR (150 MHz, DMSO-*d*_6_), *δ*: 169.1, 145.4, 143.9, 140.3, 129.9, 120.2, 109.1, 105.9, 82.2, 76.8, 76.8, 76.0, 73.7, 70.0, 61.1, 42.8, 26.7, 25.8, 18.8, 15.1. The above data are consistent with those reported in Reference [57], thus the compound is identified as 9α-hydroxyfraxinellone-9-O-β-D-glucoside.

Compound **XV**: haploperoside A (6-methoxy-7-[(2*S*,3*R*,4*S*,5*S*,6*R*)-3,4,5-trihydroxy-6-[[(2*R*,3*R*,4*R*,5*R*,6*S*)-3,4,5-trihydroxy-6-methyloxan-2-yl]oxymethyl]oxan-2-yl]oxychromen-2-one), white crystals. ESI-MS *m*/*z*: [M+H]^+^ 501.1605 (calcd for C_22_H_28_O_13_, 501.1608). ^1^H NMR (600 MHz, DMSO-*d*_6_), *δ*: 7.99 (d, *J* = 9.5 Hz, 1H), 7.31 (s, 1H), 7.15 (s, 1H), 6.35 (d, *J* = 9.4 Hz, 1H), 5.39 (d, *J* = 4.3 Hz, 1H), 5.21 (d, *J* = 4.9 Hz, 2H), 5.06 (d, *J* = 7.6 Hz, 1H), 4.67 (d, *J* = 5.4 Hz, 1H), 4.56 (d, *J* = 4.3 Hz, 1H), 4.50 (d, *J* = 1.6 Hz, 1H), 4.41 (d, J = 5.6 Hz, 1H), 3.82 (s, 4H), 3.55–3.61 (m, 2H), 3.52 (ddd, *J* = 9.2, 5.6, 3.4 Hz, 1H), 3.41 (td, *J* = 10.5, 6.4 Hz, 2H), 3.29 (s, 2H), 3.14 (s, 2H), 1.06 (d, *J* = 6.2 Hz, 3H). ^13^C NMR (150 MHz, DMSO-*d*_6_), *δ*: 160.9, 149.9, 148.8, 146.1, 144.4, 113.3, 112.4, 109.8, 103.2, 100.4, 99.8, 76.7, 75.5, 73.0, 71.8, 70.6, 70.4, 69.7, 68.3, 66.0, 56.0, 17.8. The above data are consistent with those reported in References [60,61], thus the compound is identified as haploperoside A.

Compound **XVI**: 4-hydroxy-3-methoxy-acetophenone-4-O-α-L-rhamnopyranosyl-(1→6)-β-D-glucopyranoside (1-(3-methoxy-4-(((2*S*,3*R*,4*S*,5*S*,6*R*)-3,4,5-trihydroxy-6-((((2*R*,3*R*,4*R*,5*R*,6*S*)-3,4,5-trihydroxy-6-methyltetrahydro-2*H*-pyran-2-yl)oxy)methyl)tetrahydro-2*H*-pyran-2-yl)oxy)phenyl)ethan-1-one), white powder. ESI-MS *m*/*z*: [M+Na]^+^ 497.1635 (calcd for C_21_H_30_O_12_, 497.1635). ^1^H NMR (600 MHz, DMSO-*d*_6_), *δ*: 7.59 (dd, *J* = 8.5, 2.1 Hz, 1H), 7.47 (d, *J* = 2.1 Hz, 1H), 7.13 (d, *J* = 8.5 Hz, 1H), 5.37 (s, 1H), 5.20 (s, 2H), 5.01 (d, *J* = 7.5 Hz, 1H), 4.75 (d, *J* = 5.4 Hz, 1H), 4.66 (d, *J* = 4.3 Hz, 1H), 4.54–4.59 (m, 1H), 4.52 (d, *J* = 1.6 Hz, 1H), 3.83 (s, 4H), 3.57 (q, *J* = 2.6 Hz, 1H), 3.52 (ddd, *J* = 9.4, 7.2, 1.8 Hz, 1H), 3.38–3.47 (m, 3H), 3.29 (d, *J* = 4.7 Hz, 2H), 3.17 (td, *J* = 9.3, 3.8 Hz, 1H), 3.07–3.14 (m, 1H), 2.54 (s, 3H), 1.10 (d, *J* = 6.2 Hz, 3H). ^13^C NMR (150 MHz, DMSO-*d*_6_), *δ*: 196.5, 150.5, 148.7, 131.0, 122.7, 114.4, 111.0, 100.7, 99.6, 76.8, 75.7, 73.1, 72.0, 70.7, 70.4, 69.9, 68.5, 66.6, 55.6, 26.5, 17.9. The above data are consistent with those reported in Reference [62], thus the compound is identified as 4-hydroxy-3-methoxy-acetophenone-4-O-α-L-rhamnopyranosyl-(1→6)-β-D-Glucopyranoside.

Compound **XVII**: (+)-lyoniresinol-3α-O-β-D-glucopyranoside ((2*R*,3*R*,4*S*,5*S*,6*R*)-2-(((1*S*,2*R*,3*R*)-7-hydroxy-1-(4-hydroxy-3,5-dimethoxyphenyl)-3-(hydroxymethyl)-6,8-dimethoxy-1,2,3,4-tetrahydronaphthalen-2-yl)methoxy)-6-(hydroxymethyl)tetrahydro-2*H*-pyra-n-3,4,5-triol), amorphous powder. ESI-MS *m*/*z*: [M−H]^−^ 581.2225 (calcd for C_28_H_38_O_13_, 581.2234). ^1^H NMR (600 MHz, DMSO-*d*_6_) *δ*: 6.54 (s, 1H), 6.34 (s, 2H), 4.29 (d, *J* = 5.8 Hz, 1H), 4.16 (d, *J* = 7.8 Hz, 1H), 3.76 (s, 3H), 3.67–3.71 (m, 1H), 3.64–3.67 (m, 1H), 3.64 (s, 6H), 3.48–3.52 (m, 1H), 3.38–3.44 (m, 1H), 3.33–3.36 (m, 1H), 3.28 (s, 3H), 3.24–3.27 (m, 1H), 3.11–3.18 (m, 2H), 3.03–3.10 (m, 2H), 2.98–3.03 (m, 1H), 2.62 (dd, *J* = 15.1, 4.4 Hz, 1H), 1.94–1.99 (m, 1H), 1.44–1.53 (m, 1H). ^13^C NMR (150 MHz, DMSO-*d*_6_) *δ*: 147.5 × 2, 146.9, 146.5, 137.5, 137.3, 133.3, 128.4, 124.9, 106.7, 105.9 × 2, 103.4, 76.9, 76.9, 73.5, 70.1, 69.6, 64.0, 61.1, 58.9, 56.1 × 2, 55.7, 44.4, 40.6, 40.1, 32.4. The above data are consistent with those reported in Reference [63], thus the compound is identified as (+)-lyoniresinol-3α-O-β-D-glucopyranoside.

### 3.2. Chromatographic and Mass Spectrometric Characterization

**Figure 2:**
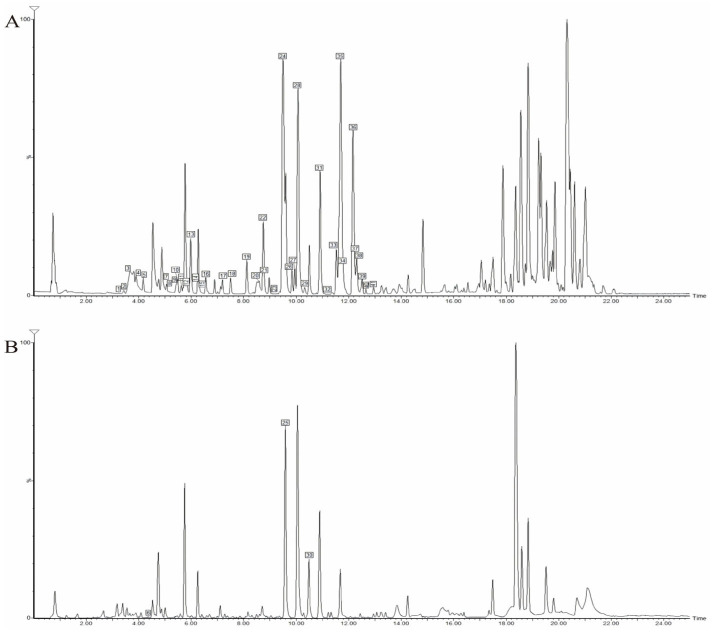
UPLC-Q-TOF-MS BPI chromatogram of DC extracted thick paste. (**A**) Positive ion mode; (**B**) Negative ion mode.

Based on the high-resolution data acquired from UPLC-Q-TOF-MS in both positive and negative ion modes, we analyzed the chemical constituents of the DC ethanolic extract. By comparing with reference standards and literature data, a total of 41 compounds were tentatively identified (Table 2), 17 of which were confirmed by comparison with reference compounds isolated in this study. The base peak intensity (BPI) chromatogram of the ethanolic extract is shown in Figure 2. The majority of these constituents were alkaloids and limonoids, with minor amounts of glycosides.

**Figure 3:**
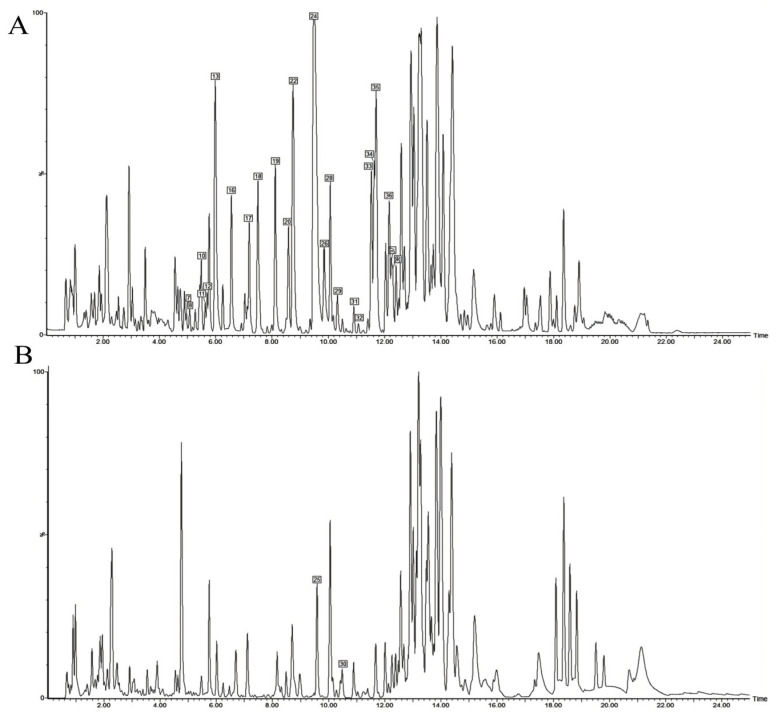
UPLC-Q-TOF-MS BPI chromatogram of transdermal sample of DC extracted thick paste. (**A**) Positive ion mode; (**B**) Negative ion mode.

We next analyzed the transdermal permeate. Its base peak intensity (BPI) chromatogram is displayed in Figure 3. A total of 26 constituents were detected. Alkaloids were the most abundant transdermal constituents, including dictamnine, γ-fagarine, isomaculosidine, and isopteleine, followed by limonoids such as limonin, evodol, and rutaevin. Analysis in positive ion mode provided more comprehensive information for constituent characterization.

### 3.3. Identification and Analysis of Representative Compounds of Alkaloids and Limonoids

Limonin and obacunone are both limonoids, with primary fragmentation pathways involving the loss of H_2_O, CO, and CO_2_, and sharing a common characteristic fragment ion at *m*/*z* 161. Limonin (28, all numbering refers to Table 2) displayed a characteristic [M+H]^+^ ion at *m*/*z* 471.2020 (C_26_H_30_O_8_; calculated: 471.2019; error: −0.21 ppm). Fragment ions included *m*/*z* 427 [M+H-CO_2_]^+^, 425 [M+H-CO-H_2_O]^+^, and 161 [M+H-C_16_H_22_O_6_]^+^. Compound 35 (obacunone) showed a protonated molecular ion peak at *m*/*z* 455.2070 [M+H]^+^ (C_26_H_30_O_7_; calculated: 455.2070; error: 0.00 ppm). Fragment ions included *m*/*z* 437 [M+H-H_2_O]^+^, 411 [M+H-CO_2_]^+^, 409 [M+H-CO-H_2_O]^+^, and 161 [M+H-C_16_H_22_O_5_]^+^ [67].

**Figure 4:**
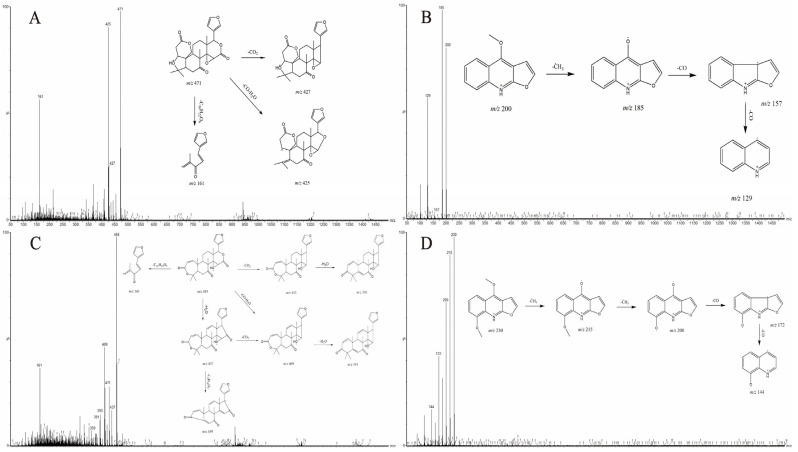
Secondary mass spectra and possible fragmentation patterns of (**A**) Limonin (**B**) Dictamnine (**C**) Obacunone, and (**D**) γ-fagarine.

Quinoline alkaloids in DC contain methoxy and carbonyl groups, exhibiting characteristic fragmentation pathways involving the loss of CO (carbonyl), CH_3_ (methyl), or combinations thereof (e.g., CO+CH_3_, 2×CO, 2×CH_3_). These constituents typically generate fragment ions at *m*/*z* 200, 201, or 202. The [M+H]^+^ peak of γ-fagarine (22) was observed at *m*/*z* 230.0817 (C_13_H_11_NO_3_; calculated: 230.0817; error: 0.00 ppm). Fragment ions included *m*/*z* 215 [M+H-CH_3_]^+^, 200 [M+H-2CH_3_]^+^, 172 [M+H-2CH_3_-CO]^+^, and 144 [M+H-2CH_3_-2CO]^+^. Dictamnine (24) exhibited a prominent [M+H]^+^ signal at *m*/*z* 200.0713 (C_12_H_9_NO_2_; calculated: 200.0712; error: −0.50 ppm) in positive ion mode. Fragment ions included *m*/*z* 185 [M+H-CH_3_]^+^, 157 [M+H-CH_3_-CO]^+^, and 129 [M+H-CH_3_-2CO]^+^ [65]. Based on characteristic fragmentation patterns and previous studies, compound 22 was identified as γ-fagarine and compound 24 as dictamnine. Figure 4 shows the specific fragmentation patterns.

### 3.4. Integrated Network Pharmacological and Molecular Docking Evaluation

#### 3.4.1. Targeted Prediction of Transdermal Constituents of DC

**Figure 5:**
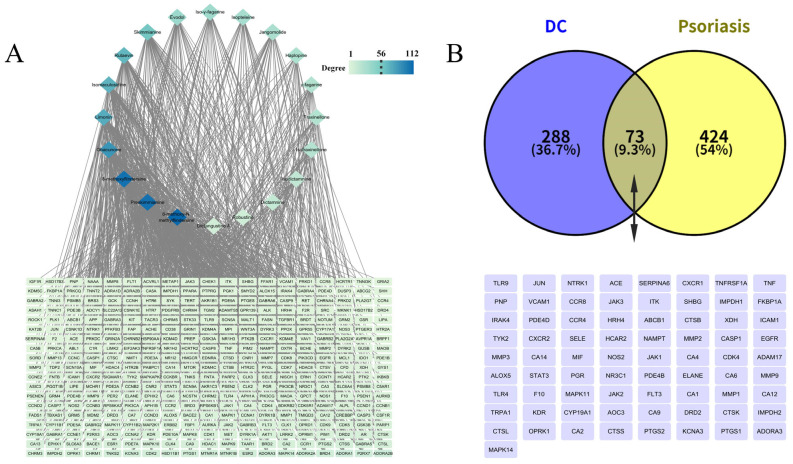
(**A**) DC “transdermal constituents-targets” network diagram; (**B**) Venn diagram of intersection targets between transdermal constituents and diseases.

Among the 26 transdermal constituents of DC, a total of 20 compounds could obtain the predicted targets. According to the predetermined screening criteria, 976 targets of transdermal constituents of DC were determined (361 targets after combining and de-weighting). The “transdermal constituent-target” interaction network was constructed with Cytoscape software (version 3.10.2; Figure 5A). In this network, diamond nodes represent transdermal constituents of DC, rectangular nodes represent constituent targets, and each edge denotes a compound-target interaction. Node degree reflects the number of edges incident to a node. A higher node degree indicates more connected compounds or targets, and thus suggesting a higher likelihood of being a key compound or target [72]. Node color intensity in the network diagram correlates with degree value, with darker colors indicating higher degrees. 8-methoxy-N-methylflindersine, preskimmianine, and 8-methoxyflindersine interacted with the largest number of target proteins, with degree values of 112, 107, and 107, respectively. Among the target proteins, ADORA2B exhibited the highest degree value (12), representing the potential key target with the highest connectivity.

Comparative analysis revealed 73 common targets between the 361 percutaneous constituent targets and 497 psoriasis-associated targets curated from DrugBank, GeneCards, TTD, and OMIM databases (Figure 5B). These shared targets, including mitogen-activated protein kinase 14 (MAPK14), adenosine A3 receptor (ADORA3), prostaglandin-endoperoxide synthase 1 (PTGS1), potassium voltage-gated channel subfamily A member 3 (KCNA3), prostaglandin-endoperoxide synthase 2 (PTGS2), represent potential therapeutic mediators of DC’s transdermal components in psoriasis management.”

#### 3.4.2. PPI Network Topology and Central Target Identification

**Figure 6:**
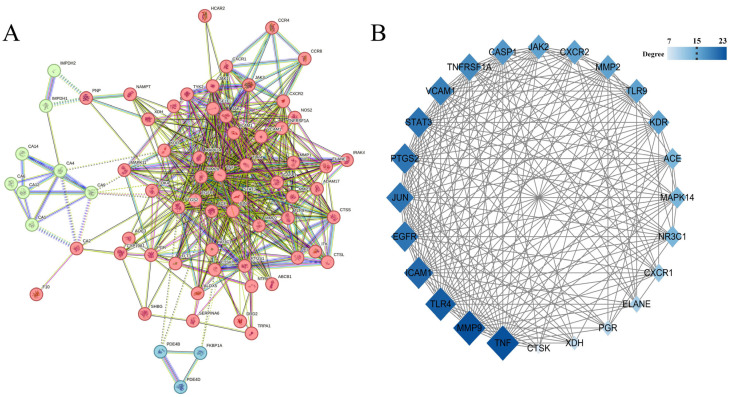
(**A**) PPI network, the colors are obtained based on k-means clustering analysis; (**B**) Core target network.

The 73 candidate targets were analyzed using the STRING platform to generate a PPI network (Figure 6A), comprising 69 interconnected nodes with 538 edges. Target networks were subjected to topological analysis and visualization in Cytoscape (version 3.10.2). Median values of node degree, closeness centrality, and betweenness centrality were used as cutoff thresholds. Nodes with values greater than or equal to the median for all three parameters were identified as core targets, resulting in 24 core targets. Network analysis revealed positive correlations between node value magnitude and both visual parameters (size and color gradient), as demonstrated in Figure 6B. Subsequently, the top five core targets were selected as target proteins for molecular docking: tumor necrosis factor (TNF), matrix metalloproteinase-9 (MMP9), Toll-like receptor 4 (TLR4), intercellular adhesion molecule-1 (ICAM-1), and epidermal growth factor receptor (EGFR).

In the TNF family, TNF-α is primarily secreted by monocytes and macrophages, while TNF-β is produced by activated T cells. TNF-α is not only a core driver of the inflammatory response but also induces the aberrant secretion of pro-inflammatory cytokines (e.g., IL-17 and IL-23) and promotes keratinocyte hyperproliferation. It critically regulates psoriasis pathogenesis [46]. TLR4 initiates MyD88-dependent signaling pathways by forming a complex with the adaptor protein MyD88, which in turn activates the nuclear factor-κB (NF-κB) canonical signaling pathway, induces the secretion of pro-inflammatory factors, and promotes the development and progression of psoriasis. NF-κB phosphorylation can directly regulate MMP9 transcriptional activation, which activates dermal vascular endothelial cells, leading to vasodilation and increased vascular permeability, thereby contributing to the formation of psoriatic lesions [73,74]. ICAM-1, a transmembrane adhesion molecule expressed on T cells and keratinocytes, mediates T cell infiltration in psoriatic lesions. Clinical studies have demonstrated elevated ICAM-1 levels in serum and lesional keratinocytes of psoriasis patients [75,76]. EGFR is a receptor tyrosine kinase widely expressed in skin tissues, serving as a key regulator of epidermal cell proliferation, differentiation, migration, and inflammation. EGFR and its ligands (e.g., TGF-α, amphiregulin, and HB-EGF) are aberrantly overexpressed and activated in the epidermis of active psoriatic lesions, indicating that EGFR-mediated hyperstimulation of keratinocytes may contribute to the development of psoriatic lesions, highlighting the important role of EGFR in psoriasis pathogenesis [77,78].

Other core targets, including JUN, STAT3, and PTGS2, are also closely associated with the pathogenesis of psoriasis. The c-Jun protein is a key transcriptional component of the activator protein-1 (AP-1) complex. Its activation regulates cell proliferation, apoptosis, and other cellular processes, and can enhance AP-1 activity to promote inflammatory responses [79,80]. PTGS2 is highly expressed in psoriatic lesions, and its encoded cyclooxygenase-2 (COX-2) participates in inflammatory responses by catalyzing the metabolism of arachidonic acid to generate prostaglandin inflammatory mediators [81]. STAT3 is a recognized potential therapeutic target in psoriasis. STAT3 activation drives Th17 cell differentiation and production of pro-inflammatory cytokines (IL-17, IL-23), critically regulating disease progression and cellular proliferation [82,83]. The core targets were shown to be essential for the efficacy of the transdermal components of DC in the treatment of psoriasis.

#### 3.4.3. Bioinformatic Analysis of Functional Annotations and Pathways

**Figure 7:**
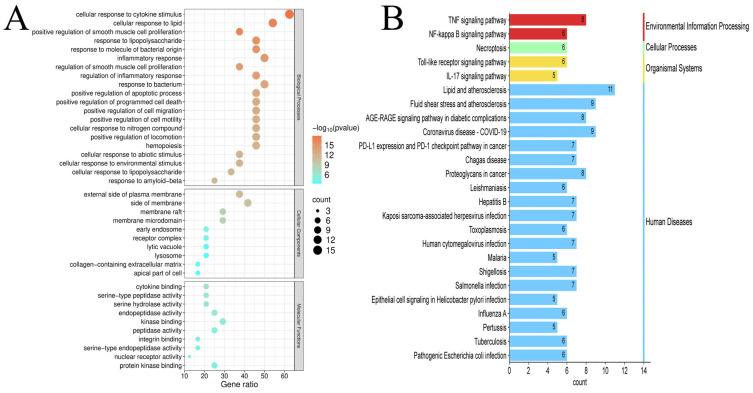
(**A**) GO analysis; (**B**) KEGG analysis.

To elucidate DC transdermal components’ anti-psoriatic mechanisms, we conducted GO/KEGG analyses on 24 core targets. GO enrichment revealed 509 significant terms (*p* < 0.01). Of these, 456 terms were associated with BPs, including “cellular response to cytokine stimulus”, “cellular response to lipid”, “positive regulation of smooth muscle cell proliferation”; 21 terms were related to CCs, including “external side of plasma membrane”, “side of membrane”, “membrane raft”; and 32 terms were associated with MFs, including “cytokine binding”, “serine-type peptidase activity”, “serine hydrolase activity”. Figure 7A displays the top-ranked terms, including 20 biological processes and 10 entries each for cellular components and molecular functions. GO enrichment analysis suggested that DC constituents might modulate inflammatory signaling pathways, down-regulate vascular smooth muscle proliferation and extracellular matrix degradation by inhibiting cellular responses to cytokines, lipid stimulation, and inflammatory stimuli. These effects may alleviate psoriatic inflammation and erythema. DC constituents may also target membrane-associated constituents, affect membrane receptor complexes, regulate cellular signal transduction, modulate enzyme activities, reduce inflammatory mediator release, and improve psoriatic symptoms.

KEGG pathway analysis identified 84 significant pathways (*p* < 0.01). The enrichment bar graph showed the top 25 pathways enriched in KEGG for 24 key genes (Figure 7B). Multiple key targets were associated with pathways, including “Lipid and Atherosclerosis”, “Fluid Shear Stress and Atherosclerosis”, “AGE-RAGE signaling pathway in diabetic complications”, “TNF signaling pathway”, and “Coronavirus disease-COVID-19”. KEGG pathway prediction suggests that DC modulates multiple immune and inflammatory pathways. Its transdermal constituents may alleviate psoriatic inflammation and keratinocyte hyperproliferation through multiple mechanisms, including inhibiting inflammatory signaling pathways (e.g., IL-17, TNF, and NF-κB pathways), regulating immune dysregulation (e.g., PD-1/PD-L1 checkpoint signaling), modulating oxidative stress (e.g., AGE-RAGE signaling), and promoting epidermal barrier repair.

Psoriasis is characterized by keratinocyte hyperproliferation, aberrant differentiation, T-cell infiltration, and increased inflammatory mediators in epidermal tissue. The TNF signaling pathway regulates inflammatory and immune responses. In the presence of pro-inflammatory signals, the expression of multiple TNF superfamily ligands and receptors is upregulated in immune cells. Binding of TNF superfamily ligands to their respective receptors on target cells initiates an intracellular signaling cascade that activates key transcription factors, including NF-κB and AP-1. This orchestrates transcription that promotes inflammation and immune effector functions [84]. The NF-κB signaling pathway is divided into canonical and noncanonical pathways. When activated, the canonical NF-κB pathway mediates responses to various external stimuli involved in inflammatory responses, immune regulation, cell proliferation, differentiation, and survival. Accumulating evidence has established a critical role of the NF-κB signaling pathway in psoriasis pathogenesis. The NF-κB noncanonical pathway plays a key role in immune cell development at multiple levels [85,86,87]. Impaired immune tolerance is considered a pathogenic factor in psoriasis and related immune-mediated diseases. The programmed cell death protein 1 (PD-1) receptor critically maintains immune tolerance through PD-L1/PD-L2 interactions, preventing immune-mediated disorders. Emerging evidence has implicated the PD-1/PD-L1 axis in psoriasis pathogenesis [88,89]. In addition, additional enriched pathways include the “AGE-RAGE signaling pathway in diabetic complications”, “Lipid and Atherosclerosis”, “Fluid Shear Stress and Atherosclerosis”, and “Coronavirus disease-COVID-19 pathway”. These findings suggest that psoriasis shares common signaling pathways with metabolic disorders, cardiovascular diseases, diabetes, and infectious diseases, indicating potential comorbidities.

#### 3.4.4. Establishment and Evaluation of the “Transdermal Component-Core Target-Key Pathway” Multidimensional Network

**Figure 8:**
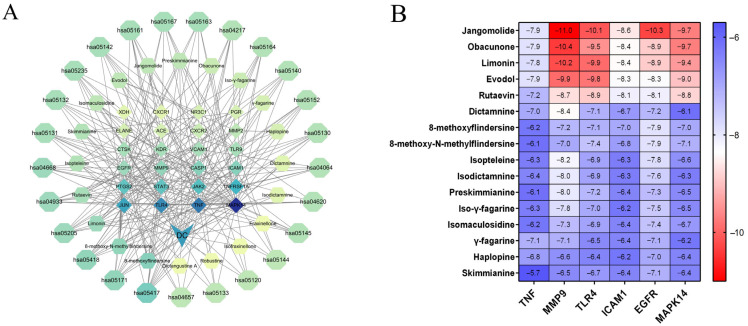
(**A**) Multidimensional network diagram of “transdermal constituents-core targets-key pathways”, color intensity indicating the interaction degree; (**B**) Molecular docking heatmap of DC transdermal constituents.

We constructed a multidimensional “component-target-pathway” network model integrating 20 transdermal components, 24 core targets, and the top 25 DC-related KEGG pathways using network pharmacology approaches (Figure 8A; see Supplementary Material Table S1 for pathway details). In the network, diamonds represent targets, hexagons represent transdermal constituents, and polygons represent KEGG pathways. Edges represent interactions between nodes, with node color intensity indicating the interaction degree. The results showed that alkaloid constituents exhibited strong associations with targets including MAPK14, PTGS2, ELANE, JAK2, and TLR4, while limonoids were associated with targets including CTSK, KDR, NR3C1, and MMP9. MAPK14 demonstrated the highest connectivity with transdermal components and pathways, qualifying it as the candidate target for docking studies. The TNF, MAPK14, TLR4, and JUN were involved in over 15 key pathways, with TNF involved in 22 pathways and MAPK14 in 21 pathways. These findings suggest that DC exerts its anti-psoriatic effects through modulation of these key targets. Dictangustine A, fraxinellone, isofraxinellone, and robustine did not exhibit associations with the 24 core targets, so the top 16 constituents were selected for molecular docking. These network pharmacological findings indicate that the transdermal constituents of DC exert therapeutic effects on psoriasis through a multi-target, multi-pathway mechanism.

#### 3.4.5. Molecular Docking Verification Analysis

The top 5 key targets and MAPK14 were verified by molecular docking with 16 transdermal constituents of DC. Structural data were obtained from the PDB for TNF (5UUI), MMP9 (4XCT), TLR4 (4G8A), ICAM1 (2OZ4), EGFR (8A2D), and MAPK14 (5ETA). These targets were identified as potential therapeutic targets for psoriasis. Molecular docking between constituents and protein receptors was performed using AutoDock Vina. A lower Vina score indicates stronger binding activity, higher molecular affinity, and a more stable complex structure. The results showed that the main transdermal constituents exhibited negative binding energies and favorable docking poses with core targets, with detailed binding energy values provided in Figure 8B. All transdermal constituents displayed good docking affinity with protein receptors (Vina score < −5.0 kcal/mol).

As shown in the thermogram (Figure 8B), limonoids, such as jangomolide, obacunone, limonin, evodol, and rutaevin, exhibited better binding affinity than alkaloids. Alkaloids generally displayed similar binding affinity, with dictamnine showing better binding than other alkaloids. These findings suggest that these constituents in DC may play key roles in improving psoriasis, potentially through interactions with key targets including TNF, MMP9, TLR4, ICAM1, EGFR, and MAPK14. These six compounds were selected for subsequent in vitro activity screening.

Jangomolide exhibited the strongest binding affinity with MMP9 among all docking results, with a binding energy of −11.0 kcal/mol (1 kcal/mol = 4.184 kJ/mol). In the molecular docking analysis, dictamnine, jangomolide, obacunone, limonin, evodol, and rutaevin exhibited stable binding with MMP9 and low binding energy. These six compound-target pairs were selected for visualization using PyMoL (version 3.1.0) software.

**Figure 9:**
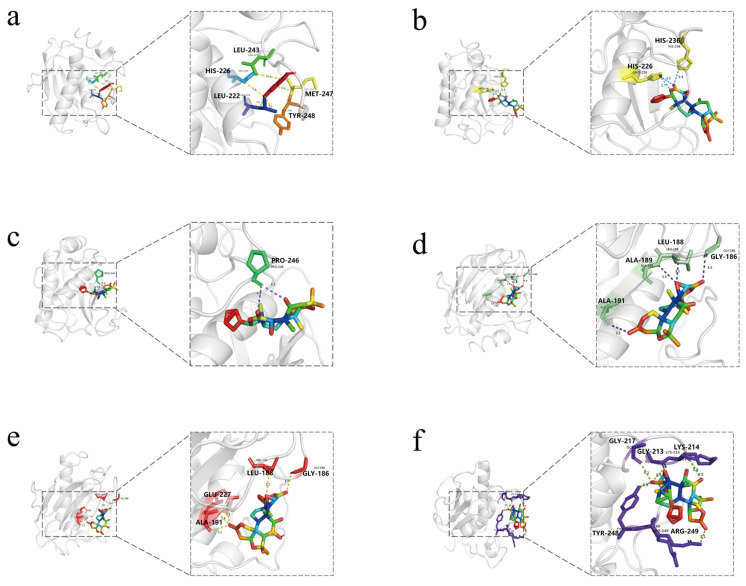
Three-dimensional Visualization models of molecular docking with MMP9 for (**a**) Dictamnine; (**b**) Jangomolide; (**c**) Obacunone; (**d**) Limonin; (**e**) Evodol; (**f**) Rutaevin.

As can be seen from the 3D model diagram (Figure 9), dictamnine forms five hydrogen bonds with residues of MMP9 between LEU243, HIS226, LEU222, TYR248, and MET247. Jangomolide forms three hydrogen bonds with residues of MMP9 between HIS236 and HIS226. Obacunone forms two hydrogen bonds with MMP9 at PRO246. Limonin forms four hydrogen bonds with residues of MMP9 between ALA191, ALA189, LEU188, and GLY186, and evodol forms four hydrogen bonds with residues of MMP9 between GLY186, LEU188, GLU227, and ALA191. rutaevin forms five hydrogen bonds with residues of MMP9 between ARG249, TYR248, GLY217, GLY213, and LYS214. The small molecules form stable complexes with the target protein’s binding pocket through a combination of hydrogen bonding and hydrophobic interactions.

As shown by the thermogram (Figure 8B), the binding energy of the transdermal ingredient to the TLR4 protein was also mostly lower than that of the other proteins, except for the lower binding energy to the MMP9 protein. These findings indicated that MMP9 and TLR4 were key core targets for the anti-psoriatic effects of DC transdermal constituents. These targets demonstrated strong functional connections to NF-κB signaling, underscoring the central role of this pathway in psoriasis pathogenesis.

### 3.5. Analysis of In Vitro Test Results

#### 3.5.1. Effect of TNF-α on HaCaT Cells

The HaCaT cell line is a human immortalized epidermal keratinocyte line, which has similar proliferation and differentiation characteristics to epidermal stem cells. It is widely used in skin disease research and is a suitable cell line for establishing psoriatic cell models. TNF-α, a key inflammatory cytokine in psoriasis, drives both keratinocyte proliferation and inflammatory cascades, mirroring disease pathology [46,90]. Therefore, TNF-α is commonly used to construct psoriatic cell models. Wang et al. [91] induced a psoriasis-like HaCaT cell model with 20 ng/mL TNF-α and found that genistein effectively inhibited abnormal proliferation of HaCaT cells and expression of inflammatory factors. In another study, Wang et al. [10] established a psoriasis-like HaCaT cell model using 100 ng/mL TNF-α, demonstrating that tanshinone I targets both inflammatory pathways and keratinocyte differentiation to ameliorate psoriasis-like dermatitis.

**Figure 10:**
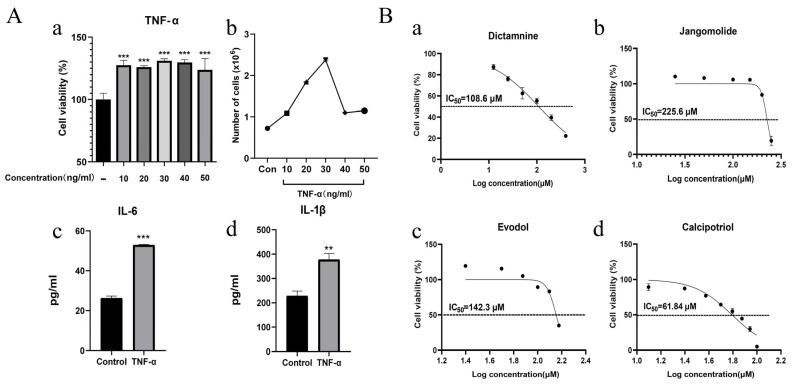
(**A**) Establishment of psoriatic cell model; (**a**) TNF-α regulation of keratinocyte proliferation; (**b**) Cell counts of HaCaT cells treated with different concentrations of TNF-α for 24 h detected by direct cell counting; (**c**) IL-6 expression modulated by 30 ng/mL TNF-α; (**d**) IL-1β expression modulated by 30 ng/mL TNF-α. (**B**) Effects of (**a**) Dictamnin; (**b**) Jangomolide; (**c**) Evodol; (**d**) Calcipotriol on normal cells and their IC_50_ values. ** *p* < 0.01 (vs. normal), *** *p* < 0.001 (vs. normal).

To mimic psoriatic conditions, HaCaT cells were stimulated with TNF-α (10–50 ng/mL, 24 h). We first evaluated its effect on proliferation via cell viability assays. TNF-α treatment (10–50 ng/mL) significantly enhanced HaCaT cell viability versus untreated controls, demonstrating a hyperproliferative response (Figure 10(Aa)). Compared with untreated HaCaT cells (cell count: 0.72 ± 0.02 × 10^6^ cells/mL), treatment with 10–50 ng/mL TNF-α significantly increased cell counts to 1.08 ± 0.02 × 10^6^, 1.85 ± 0.01 × 10^6^, 2.38 ± 0.02 × 10^6^, 1.10 ± 0.01 × 10^6^, and 1.14 ± 0.03 × 10^6^ cells/mL, respectively (Figure 10(Ab)). Among these, the 30 ng/mL TNF-α treatment produced the most potent growth-promoting effects on HaCaT cells. Therefore, the selected concentration was used to measure inflammatory mediators. Treated cells demonstrated markedly elevated IL-6 (Figure 10(Ac)) and IL-1β (Figure 10(Ad)) expression levels. These results indicated that cell proliferation and inflammatory responses were upregulated, confirming the successful establishment of the psoriatic cell model. Subsequently, 30 ng/mL TNF-α was used for subsequent modeling experiments.

#### 3.5.2. Drug Concentration Screening

**Figure 11:**
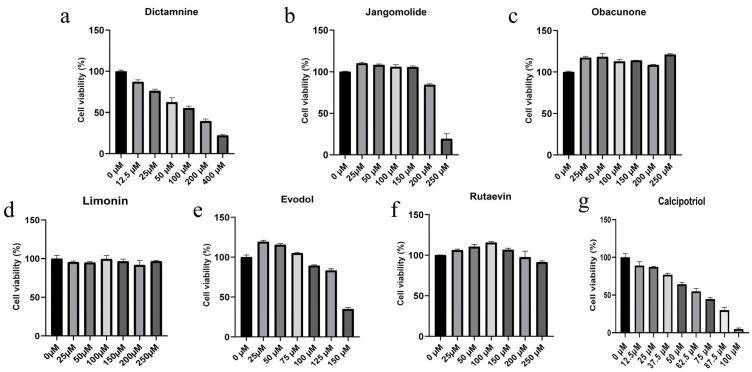
Cytotoxic effects of different compounds on normal HaCaT Cells: (**a**) Dictamnine; (**b**) Jangomolide; (**c**) Obacunone; (**d**) Limonin; (**e**) Evodol; (**f**) Rutaevin; (**g**) Calcipotriol.

In HaCaT cells, different concentrations of dictamnine, jangomolide, obacunone, limonin, evodol, rutaevin, and calcipotriol (positive control) were added for intervention based on the compounds’ physicochemical properties to screen appropriate concentrations for subsequent experiments. The results indicated that the viability of HaCaT cells was influenced by compound specificity and concentration. Dictamnine demonstrated concentration-dependent inhibition of HaCaT cell viability in CCK-8 assays, with a calculated IC_50_ of 108.6 μM (24 h treatment; Figure 10(Ba)). Jangomolide inhibited HaCaT cell viability at higher concentrations, with a fitted IC_50_ value of 225.6 μM after 24 h of treatment (Figure 10(Bb)). Evodol exhibited a fitted IC_50_ value of 142.3 μM after 24 h of treatment (Figure 10(Bc)), while calcipotriol showed a fitted IC_50_ value of 61.84 μM (Figure 10(Bd)). Obacunone, limonin, and rutaevin exhibited no cytotoxic effects at the selected concentrations. Following viability screening (Figure 11), subsequent experiments employed compound concentrations of 50, 25, and 12.5 μM

#### 3.5.3. Inhibitory Effects of Different Compounds on Abnormal HaCaT Cell Proliferation

**Figure 12:**
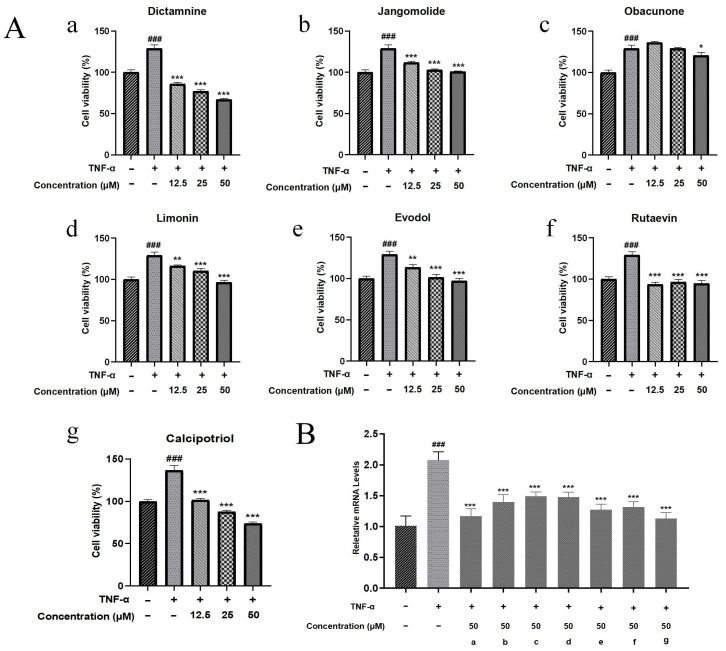
(**A**) Cellular activity against abnormal proliferation: (**a**) Dictamnine; (**b**) Jangomolide; (**c**) Obacunone; (**d**) Limonin; (**e**) Evodol; (**f**) Rutaevin; (**g**) Calcipotriol. (**B**) Inhibitory effects on Ki67 mRNA expression; (a) Dictamnine; (b) Jangomolide; (c) Obacunone; (d) Limonin; (e) Evodol; (f) Rutaevin; (g) Calcipotriol. Statistical comparisons: ### *p* < 0.001 (vs. normal); * *p* < 0.05, ** *p* < 0.01, *** *p* < 0.001 (vs. model).

Psoriasis pathogenesis is characterized by keratinocyte hyperproliferation concurrent with inflammatory activation [92]. Therefore, drugs that inhibit hyperproliferation and exhibit anti-inflammatory effects have therapeutic potential for psoriasis. The CCK-8 assay was used to quantify metabolically active cells as a proliferation indicator. In this study, we evaluated six transdermal compounds (dictamnine, jangomolide, obacunone, limonin, evodol, rutaevin) and calcipotriol on TNF-α-stimulated HaCaT cells at 12.5, 25, and 50 μM (Figure 12A). The normal group received no TNF-α treatment, while the model group was treated with TNF-α alone. Metabolic activity was significantly higher in the model group versus controls (*p* < 0.001), confirming TNF-α-mediated hyperproliferation. Compared with the model group, dictamnine, jangomolide, limonin, evodol, rutaevin, and calcipotriol significantly reduced TNF-α-induced abnormal cell proliferation at 12.5, 25, and 50 μM (*p* < 0.01). Obacunone exhibited marginal inhibitory activity at 50 μM (*p* < 0.05). Across all tested concentrations, dictamnine inhibited cell viability significantly more effectively than the other compounds, with an efficacy comparable to calcipotriol (positive control). At high and low concentrations, dictamnine inhibited aberrant cell proliferation by 48% and 33%, respectively. The inhibition rates for calcipotriol were 45% and 25% at high and low concentrations, respectively. In contrast, the remaining compounds (jangomolide, limonin, evodol, rutaevin) exhibited only approximately 20% inhibition even at high concentrations.

#### 3.5.4. Modulation of Ki67 Expression by Various Compounds in a Psoriasis-like HaCaT Model

As shown in Section 3.5.3, all tested compounds attenuated TNF-α-induced abnormal increases in cell viability to varying degrees, with significant differences observed at 50 μM. Therefore, Ki67 mRNA expression was measured for each compound at this concentration. Ki67 is a nuclear protein associated with cell proliferation, expressed during the cell proliferation cycle, and reliably reflects the cell proliferation status in psoriasis [93]. The model group exhibited significantly higher Ki67 mRNA expression compared to normal controls (*p* < 0.001). Compared with the TNF-α treatment alone, all compounds reduced Ki67 mRNA expression to varying degrees, with significant differences (*p* < 0.001). Among the transdermal compounds, dictamnine exhibited the greatest reduction, approaching the positive control level (Figure 12B).

#### 3.5.5. Effects of Different Compounds on Inflammatory Factor Expression

**Figure 13:**
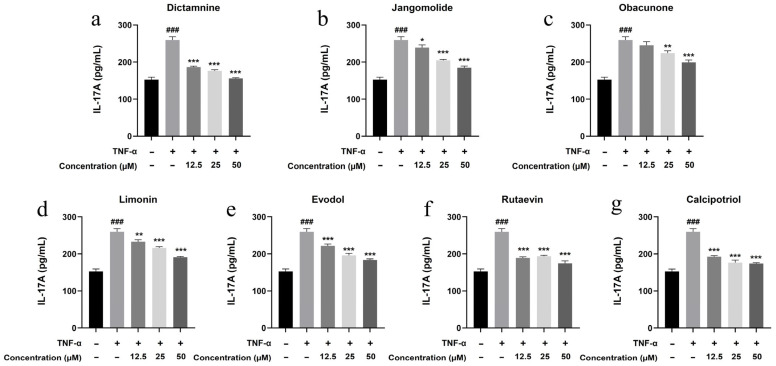
Suppression of IL-17A production by various compounds: (**a**) Dictamnine; (**b**) Jangomolide; (**c**) Obacunone; (**d**) Limonin; (**e**) Evodol; (**f**) Rutaevin; (**g**) Calcipotriol. Statistical comparisons: ### *p* < 0.001 (vs. normal); * *p* < 0.05, ** *p* < 0.01, *** *p* < 0.001 (vs. model).

**Figure 14:**
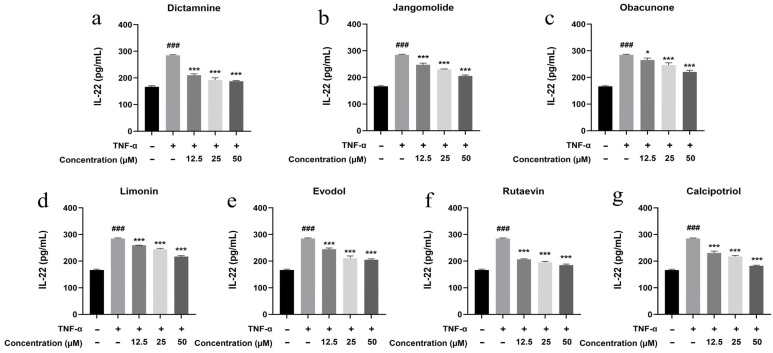
Suppression of IL-22 production by various compounds: (**a**) Dictamnine; (**b**) Jangomolide; (**c**) Obacunone; (**d**) Limonin; (**e**) Evodol; (**f**) Rutaevin; (**g**) Calcipotriol. Statistical comparisons: ### *p* < 0.001 (vs. normal); * *p* < 0.05, *** *p* < 0.001 (vs. model).

**Figure 15:**
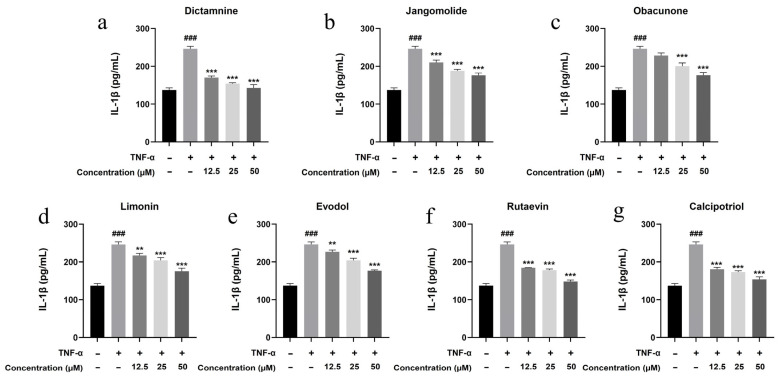
Suppression of IL-1β production by various compounds: (**a**) Dictamnine; (**b**) Jangomolide; (**c**) Obacunone; (**d**) Limonin; (**e**) Evodol; (**f**) Rutaevin; (**g**) Calcipotriol. Statistical comparisons: ### *p* < 0.001 (vs. normal); ** *p* < 0.01, *** *p* < 0.001 (vs. model).

**Figure 16:**
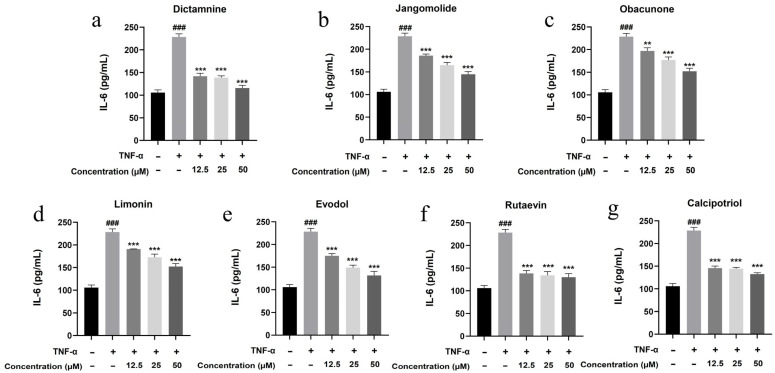
Suppression of IL-6 production by various compounds: (**a**) Dictamnine; (**b**) Jangomolide; (**c**) Obacunone; (**d**) Limonin; (**e**) Evodol; (**f**) Rutaevin; (**g**) Calcipotriol. Statistical comparisons: ### *p* < 0.001 (vs. normal) ** *p* < 0.01, *** *p* < 0.001 (vs. model).

**Figure 17:**
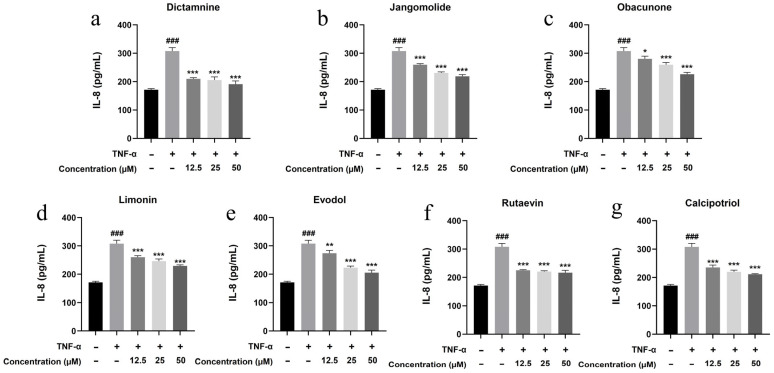
Suppression of IL-8 production by various compounds: (**a**) Dictamnine; (**b**) Jangomolide; (**c**) Obacunone; (**d**) Limonin; (**e**) Evodol; (**f**) Rutaevin; (**g**) Calcipotriol. Statistical comparisons: ### *p* < 0.001 (vs. normal); * *p* < 0.05, ** *p* < 0.01, *** *p* < 0.001 (vs. model).

Based on the TNF, NF-κB, IL-17, and Toll-like receptor signaling pathways identified through network pharmacology analysis, and considering the pathogenesis of psoriasis, the inflammatory factors IL-17A, IL-22, IL-1β, IL-6, and IL-8 were selected for detection. Keratinocytes can initiate inflammatory processes upon TNF-α stimulation, producing cytokines including IL-17A, IL-22, IL-1β, IL-6, and IL-8, which are associated with psoriasis pathogenesis [94,95,96,97,98,99]. TNF-α stimulation markedly elevated IL-17A, IL-22, IL-1β, IL-6, and IL-8 expression in HaCaT cells (*p* < 0.001; Figure 13, Figure 14, Figure 15, Figure 16 and Figure 17). All test compounds significantly reduced the levels of various inflammatory factors across tested concentrations (12.5–50 μM; *p* < 0.001; Figure 13, Figure 14, Figure 15, Figure 16 and Figure 17). Among them, dictamnine and rutaevin exhibited similar inhibitory effects, comparable to the positive control (calcipotriol), and showed excellent anti-inflammatory activity even at low concentrations. Jangomolide, limonin, obacunone, and evodol displayed comparable inhibitory efficacy. Overall, obacunone showed the weakest anti-inflammatory effect, while dictamnine exhibited the most potent anti-inflammatory activity. Among all inflammatory factors examined, dictamnine produced the most substantial suppression of IL-6, reducing its levels by 49% at high concentration and 38% at low concentration. In comparison, calcipotriol led to reductions of 42% and 36%, respectively. IL-8 was the second most markedly inhibited cytokine, with dictamnine treatment resulting in decreases of 38% (high) and 32% (low). Conversely, calcipotriol reduced IL-8 levels by 31% and 24% at the corresponding concentrations.

Based on the antiproliferative and anti-inflammatory results, all transdermal compounds significantly inhibited abnormal keratinocyte proliferation, with dictamnine exhibiting the most potent effects. The compounds showed good anti-inflammatory activity and effectively reduced levels of the inflammatory factors IL-17A, IL-22, IL-1β, IL-6, and IL-8. Among these, dictamnine and rutaevin displayed better inhibitory effects than other compounds, comparable to calcipotriol (positive control). These six compounds show therapeutic potential for psoriasis, with dictamnine showing the best antiproliferative and anti-inflammatory effects, thus establishing a foundation for developing new anti-psoriatic drugs.

## 4. Conclusions

In this study, 41 constituents of DC were identified by UPLC-Q-TOF-MS (17 chemical components isolated in this paper were used as reference substances for comparison). A total of 26 constituents were found to penetrate the skin, most of which were alkaloids and limonoids, indicating these compounds might more easily penetrate the skin to exert therapeutic effects. Network pharmacology analysis showed that 20 transdermal constituents were associated with potential therapeutic targets for psoriasis, including TNF, MMP9, TLR4, ICAM1, EGFR, and MAPK14. Functional enrichment analysis (GO/KEGG) revealed that DC’s anti-psoriatic activity involved modulation of diverse immune-inflammatory pathways. Using molecular docking to simulate ligand-target affinity, we selected six transdermal constituents with anti-psoriatic potential: dictamnine, jangomolide, obacunone, limonin, evodol, and rutaevin. In vitro anti-proliferation assays and inflammatory factor detection further confirmed that these compounds effectively inhibited abnormal cell proliferation and reduced inflammatory factor levels. Based on the evaluation of multiple indicators for each compound, dictamnine was confirmed to have the most promising anti-psoriatic effect and demonstrated potential as a candidate drug for psoriasis treatment. These results provide a pharmacodynamic material basis for the development of external preparations of DC for treating psoriasis, offer a novel research approach for exploring the pharmacodynamic material basis of external drugs, and present a new perspective for the development of external traditional Chinese medicine preparations against psoriasis.

## Figures and Tables

**Table 1:** Primer sequences used in this study.

Primer Name	Forward	Reverse
Actin	TCCTCCTGAGCGCAAGTACTCC	CATACTCCTGCTTGCTGATCCAC
Ki67	TCCAGACACCAGACCACACTGA	GCCGCCTCCTTGTGCTTGTT

**Table 2:** UPLC-Q-TOF-MS characterization of chemical components in DC ethanol extract and its transdermal permeants.

No.	RT (min)	Compounds	Molecular Formula	Ion Mode	Theoretical value (*m*/*z*)	Actual value (*m*/*z*)	Error (ppm)	Fragment Ions (*m*/*z*)	Transdermal	Structure Type	References
1	3.40	4-hydroxy-3-methoxy-acetophenone-4-O-α-L-rhamnopyranosyl-(1→6)-β-D-glucopyranoside *	C_21_H_30_O_12_	[M+Na]^+^	497.1635	497.1635	0.00	293, 167	No	acetophenone glycosides	*
2	3.47	Haploperoside A *	C_22_H_28_O_13_	[M+H]^+^	501.1608	501.1605	0.60	355, 193	No	coumarin glycosides	*
3	3.67	Dictamalkoside A *	C_18_H_19_NO_8_	[M+H]^+^	378.1189	378.1190	−0.26	340, 322, 216	No	alkaloid glycosides	*
4	3.92	3-[1β-hydroxy-2-(β-D-glucopyranosyloxy)ethyl)-4-methoxy-2(1H)-quinolinone *	C_18_H_23_NO_9_	[M+H]^+^	398.1451	398.1450	0.25	340, 322, 218	No	alkaloid glycosides	*
5	4.17	3-(2-hydroxy-3methyl-3-buten-1yl)-4-methoxy-1methyl-2(1H)quinolinone	C_16_H_19_NO_3_	[M+H]^+^	274.1443	274.1440	1.09	256, 226, 202, 186, 172, 144	No	QOA	[64]
6	4.47	(+)-lyoniresinol-3a-O-β-D-glucopyranoside *	C_28_H_38_O_13_	[M−H]^−^	581.2234	581.2225	1.55	384, 295	No	lignan glycosides	*
7	4.96	(+)-cis-7,8dimethoxymyrtopsine	C_17_H_21_NO_6_	[M+H]^+^	336.1447	336.1447	0.00	318, 290, 276, 264, 249, 234, 220, 164	Yes	DFQA	[64]
8	5.07	Dictangustine A	C_12_H_9_NO_3_	[M+H]^+^	216.0661	216.0661	0.00	201, 188, 160, 144, 134, 117	Yes	FQA	[64]
9	5.34	Acetovanillone *	C_9_H_10_O_3_	[M+H]^+^	167.0708	167.0708	0.00	141, 128	No	acetophenone derivatives	*
10	5.48	Isodictamdiol C *	C_15_H_18_O_5_	[M+K]^+^	301.1052	301.1045	2.32	261, 243, 215	Yes	L	*
11	5.63	Dasycarine D	C_17_H_21_NO_5_	[M+H]^+^	320.1498	320.1500	−0.62	302, 287, 272, 254, 248, 216, 188, 175	Yes	QOA	[64]
12	5.69	Platydesmine or isomers	C_15_H_17_NO_3_	[M+H]^+^	260.1287	260.1285	0.77	242, 210, 188, 130, 103	Yes	DFQA	[64]
13	5.98	Isodictamnine	C_12_H_9_NO_2_	[M+H]^+^	200.0712	200.0711	0.50	185, 157, 129	Yes	FQA	[65]
14	6.04	Dictamdiol B *	C_15_H_18_O_5_	[M+K]^+^	301.1052	301.1051	0.33	261, 243, 215	No	L	*
15	6.43	9α-hydroxyfraxinellone-9-O-β-D-glucoside *	C_20_H_26_O_9_	[M+H]^+^	411.1655	411.1655	0.00	291, 249, 231	No	limonoid glycosides	*
16	6.56	Iso-γ-fagarine	C_13_H_11_NO_3_	[M+H]^+^	230.0817	230.0816	0.43	215, 187, 172	Yes	FQA	[65]
17	7.20	Haplopine	C_13_H_11_NO_4_	[M+H]^+^	246.0766	246.0765	0.41	231, 216, 202, 188, 184, 160, 156, 132	Yes	FQA	[64]
18	7.50	Isopteleine	C_13_H_11_NO_3_	[M+H]^+^	230.0817	230.0817	0.00	215, 187, 172	Yes	FQA	[65]
19	8.12	Isomaculosidine	C_14_H_13_NO_4_	[M+H]^+^	260.0923	260.0923	0.00	245, 217, 202, 187, 174	Yes	FQA	[65]
20	8.58	Skimmianine	C_14_H_13_NO_4_	[M+H]^+^	260.0923	260.0923	0.00	245, 227, 216, 202, 199, 184, 174	Yes	FQA	[64]
21	8.63	Kihadanin A	C_26_H_30_O_9_	[M+H]^+^	487.1968	487.1969	−0.21	469, 441, 423, 175	No	L	[56]
22	8.75	γ-fagarine *	C_13_H_11_NO_3_	[M+H]^+^	230.0817	230.0817	0.00	215, 200, 172, 144	Yes	FQA	[65]
23	9.06	Kihadanin B	C_26_H_30_O_9_	[M+H]^+^	487.1968	487.1967	0.21	469, 441, 425, 367, 161	No	L	[56]
24	9.50	Dictamnine *	C_12_H_9_NO_2_	[M+H]^+^	200.0712	200.0713	−0.50	185, 157, 129	Yes	FQA	[65]
25	9.59	Rutaevin *	C_26_H_30_O_9_	[M−H]^−^	485.1812	485.1811	0.21	397, 383	Yes	L	[48,66]
26	9.86	Robustine	C_12_H_9_NO_3_	[M+H]^+^	216.0661	216.0657	1.85	201, 173, 145, 117	Yes	FQA	[64]
27	9.92	Fraxinellonone *	C_14_H_14_O_4_	[M+H]^+^	247.0970	247.0966	1.62	229, 216, 185	No	L	*
28	10.07	Limonin *	C_26_H_30_O_8_	[M+H]^+^	471.2019	471.2020	−0.21	427, 425, 161	Yes	L	[37,48,67]
29	10.32	8-methoxyflindersine	C_15_H_15_NO_3_	[M+H]^+^	258.1130	258.1129	0.39	243, 240, 228, 216, 204, 106	Yes	PQA	[64]
30	10.48	Evodol *	C_26_H_28_O_9_	[M−H]^−^	483.1655	483.1661	−1.24	421, 395, 161	Yes	L	[17,37]
31	10.91	Jangomolide *	C_26_H_28_O_8_	[M+H]^+^	469.1862	469.1862	0.00	317, 187, 161	Yes	L	*
32	11.08	2-hydroxy-4methoxy-3-(3′ methyl-2′ butenyl)-quinolin	C_15_H_17_NO_2_	[M+H]^+^	244.1338	244.1337	0.40	202, 200, 188, 186, 173, 160, 134, 91	Yes	QOA	[64,68,69]
33	11.53	2,6-dihydro-2,2,7-trimethyl-5H-pyrano [3,2-c] quinolin-5-one	C_15_H_15_NO_2_	[M+H]^+^	242.1181	242.1180	0.41	227, 224, 212, 200, 188, 144, 106	Yes	PQA	[64]
34	11.63	Preskimmianine *	C_17_H_21_NO_4_	[M+H]^+^	304.1549	304.1546	0.99	248, 233, 216, 188, 175, 162, 146, 132	Yes	QOA	[64]
35	11.69	Obacunone *	C_26_H_30_O_7_	[M+H]^+^	455.2070	455.2070	0.00	437, 411, 409, 393, 391, 359, 161	Yes	L	[17,67,70]
36	12.16	Fraxinellone *	C_14_H_16_O_3_	[M+H]^+^	233.1178	233.1176	0.86	215, 187, 128	Yes	L	[56]
37	12.24	8-methoxy-N-methylflindersine	C_16_H_17_NO_3_	[M+H]^+^	272.1287	272.1288	−0.37	257, 242, 230	Yes	PQA	[64]
38	12.30	Isofraxinellone	C_14_H_16_O_3_	[M+H]^+^	233.1178	233.1178	0.00	215, 187, 128	Yes	L	[56]
39	12.50	Calodendrolide or isomers	C_15_H_16_O_4_	[M+H]^+^	261.1127	261.1127	0.00	182, 169, 141, 128	No	L	[69,71]
40	12.65	7α-obacunyl acetate	C_28_H_34_O_8_	[M+H]^+^	499.2332	499.2334	−0.40	439, 258, 202	No	L	[56]
41	12.95	N-metilatanina	C_16_H_19_NO_2_	[M+H]^+^	258.1494	258.1494	0.00	216, 202, 172, 144, 115	No	QOA	[64]

* compounds aligned against controls; FQA, furoquinoline alkaloids; DFQA, dihydrofuroquinoline alkaloids; PQA, pyranoquinolinone alkaloids; QOA, quinol-2-one alkaloids; L, Limonoids.

## Data Availability

Data is contained within the article and Supplementary Materials.

## Supplementary Materials

The following supporting information can be downloaded at https://www.mdpi.com/article/10.3390/pharmaceutics17091195/s1, Figures S1–S34: Compound ^1^H and ^13^C NMR spectrum; Table S1: The top 25 KEGG pathways.
