# Supplementary material for: Anti-Psoriatic Pharmacodynamic Material Basis of Dictamni Cortex Based on Transdermal Constituents Group
Source: Pharmaceutics. 2025 Sep 14;17(9):1195. doi: 10.3390/pharmaceutics17091195 (PMC12473181; doi:10.3390/pharmaceutics17091195)
Supplement: Supplementary file 1 [file pharmaceutics-17-01195-s001.zip › pharmaceutics-3841507-supplementary.pdf]

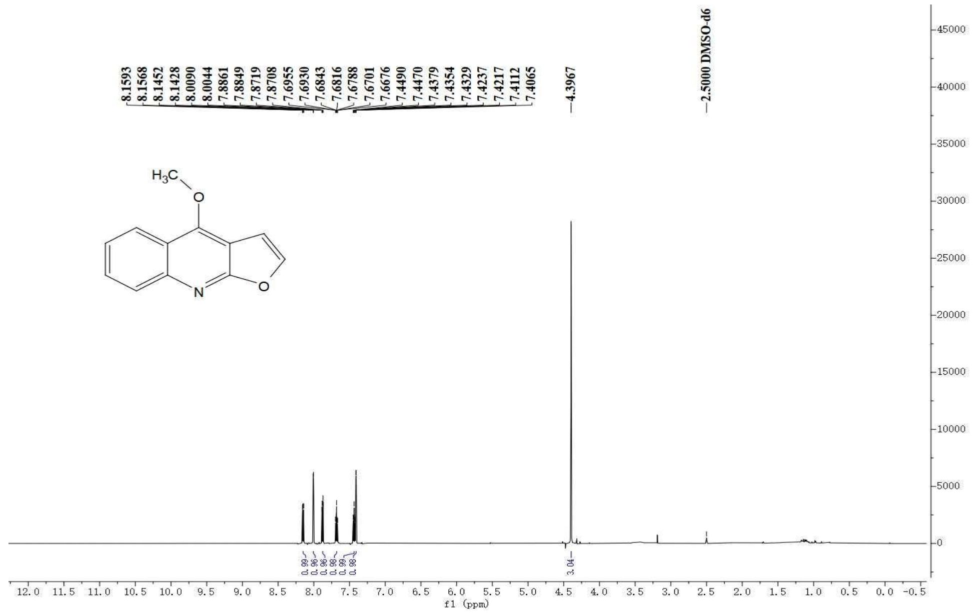

Figure S1. Compound I ^{1}H NMR spectrum.

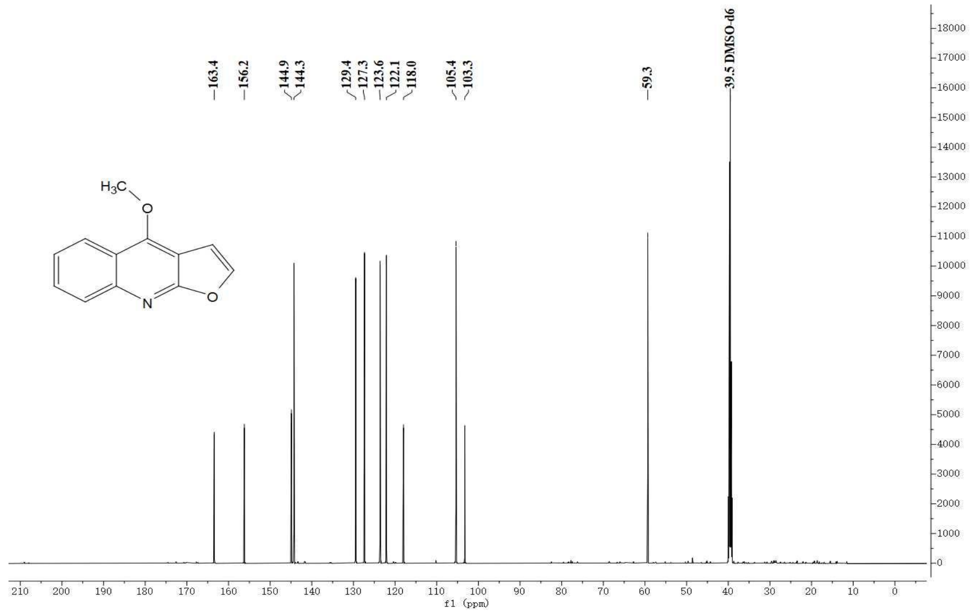

Figure S2. Compound I ^{13}C NMR spectrum.

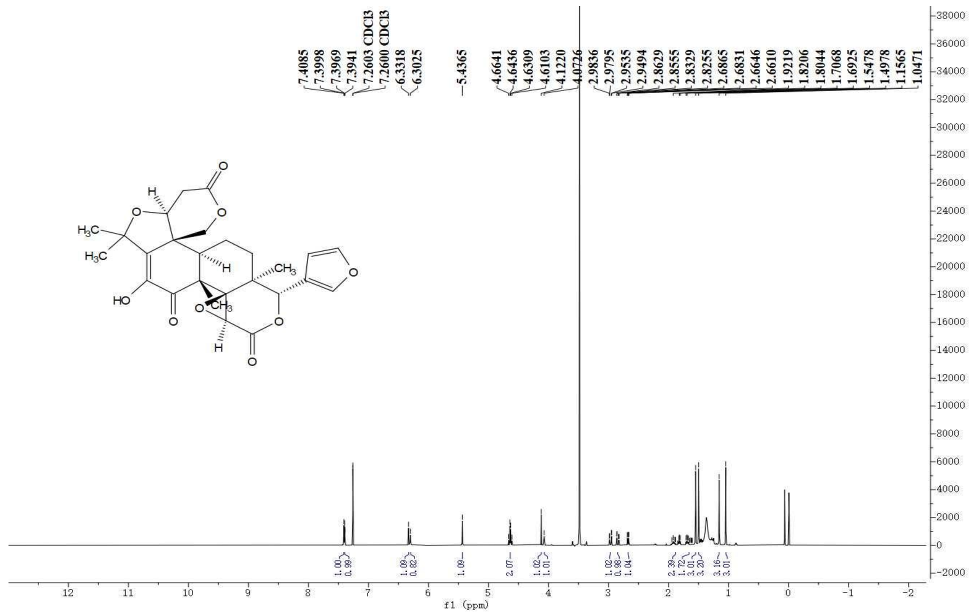

Figure S3. Compound II ¹H NMR spectrum.

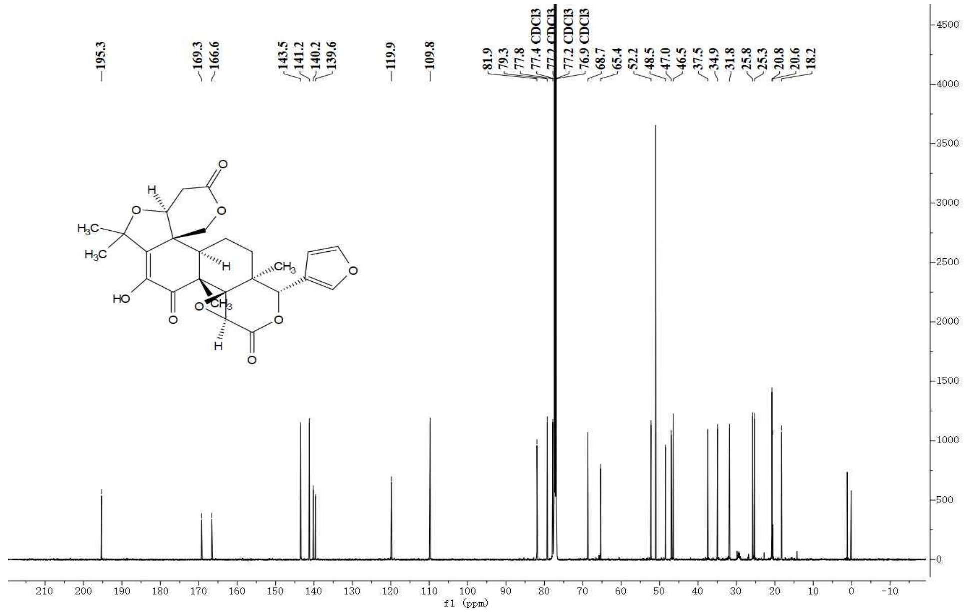

Figure S4. Compound II ¹³C NMR spectrum.

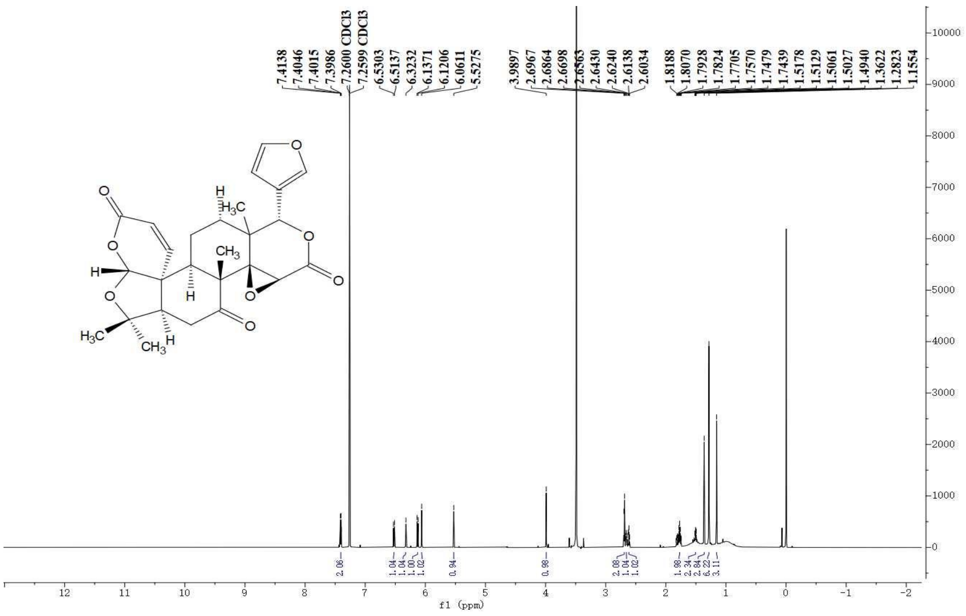

Figure S5. Compound III ^{1}H NMR spectrum.

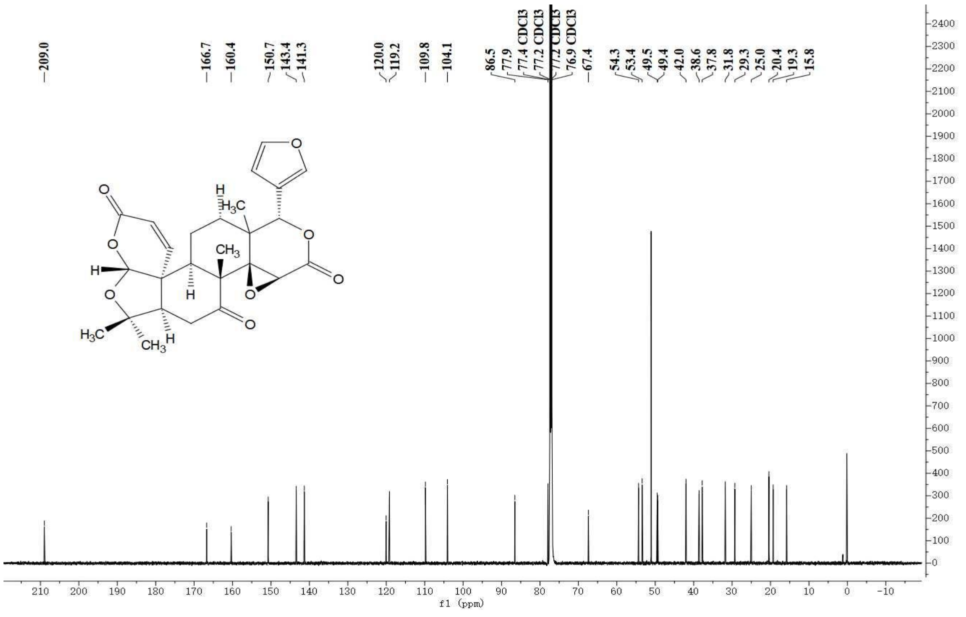

Figure S6. Compound III ^{13}C NMR spectrum.

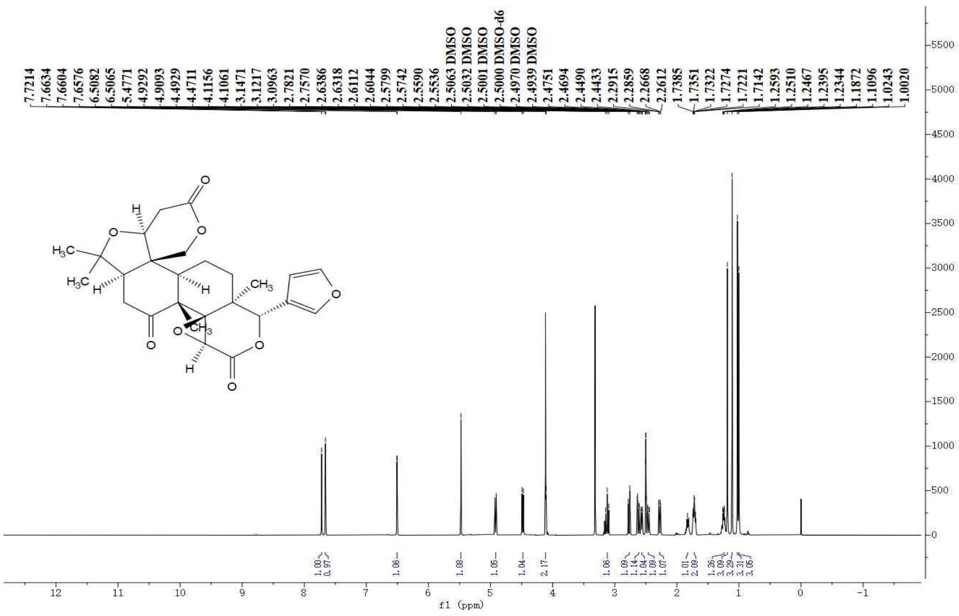

Figure S7. Compound IV ^{1}H NMR spectrum.

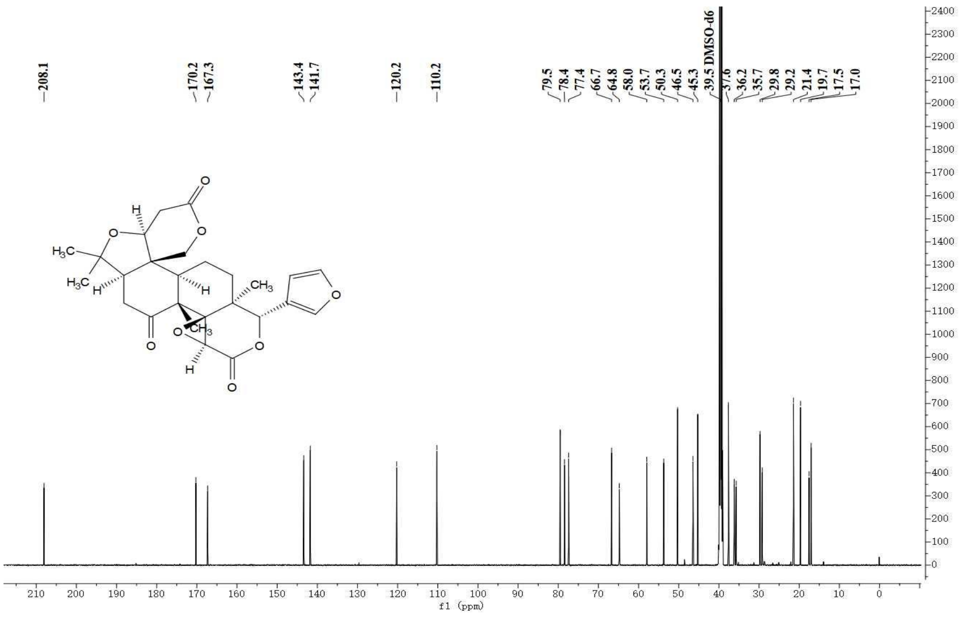

Figure S8. Compound IV ^{13}C NMR spectrum.

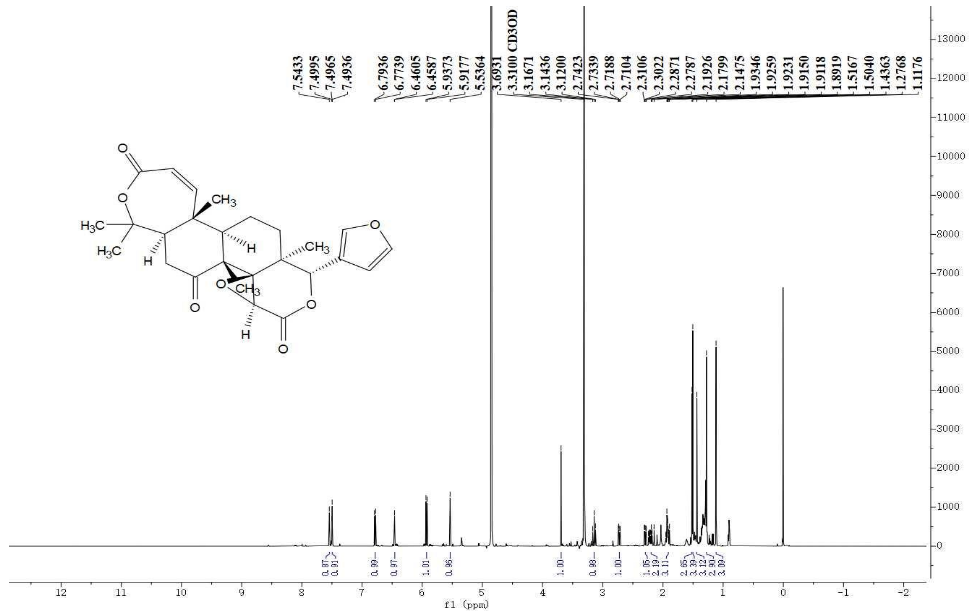

Figure S9. Compound V ^{1}H NMR spectrum.

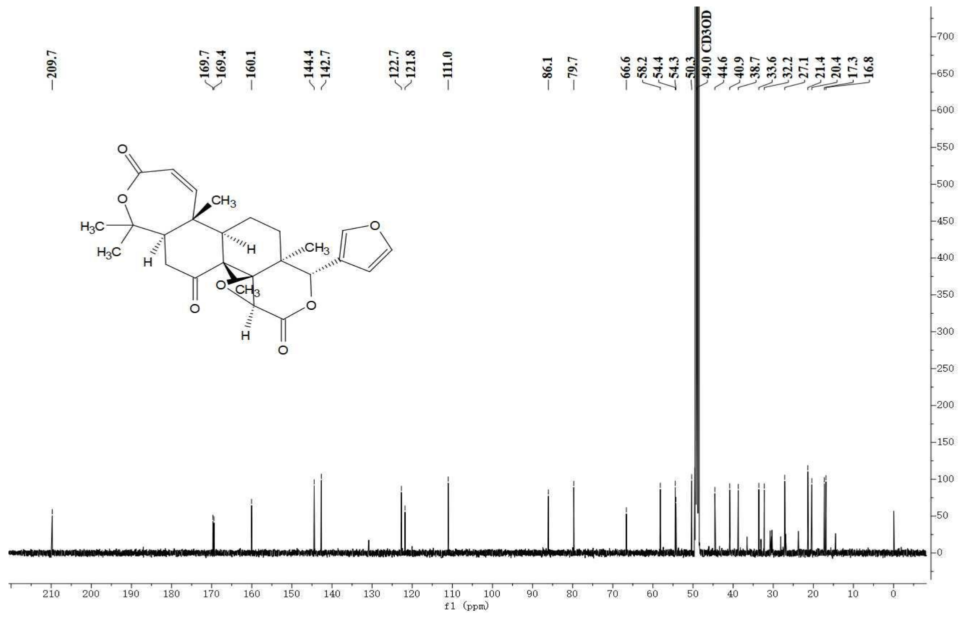

Figure S10. Compound V ^{13}C NMR spectrum.

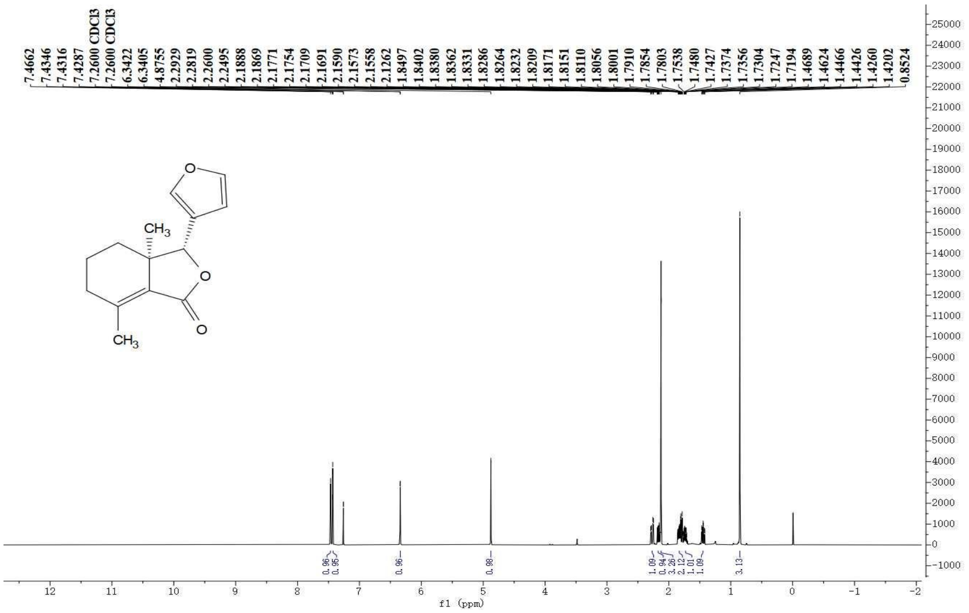

Figure S11. Compound VI ^{1}H NMR spectrum.

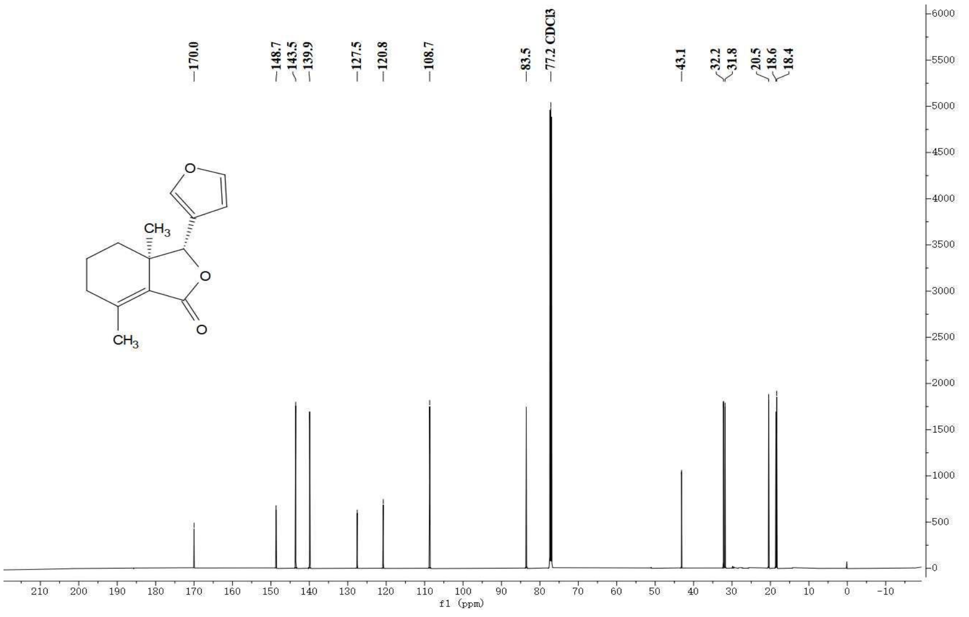

Figure S12. Compound VI ^{13}C NMR spectrum.

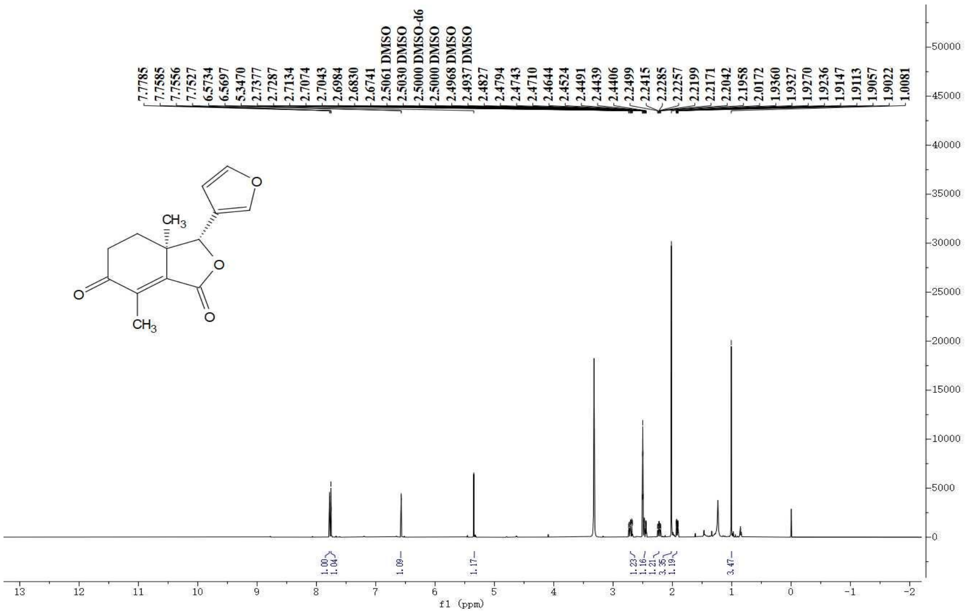

Figure S13. Compound VII ^{1}H NMR spectrum.

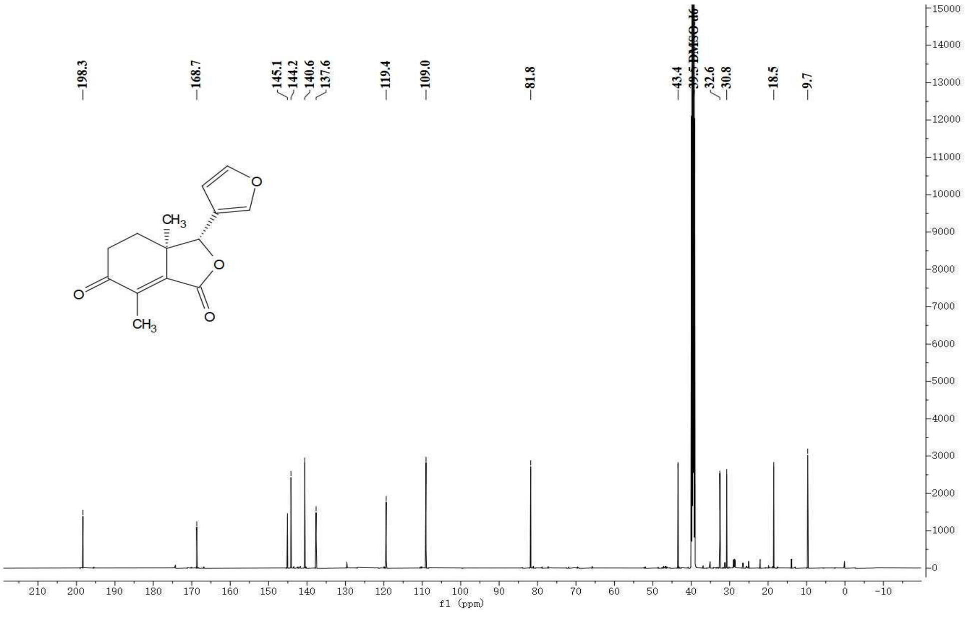

Figure S14. Compound VII ^{13}C NMR spectrum.

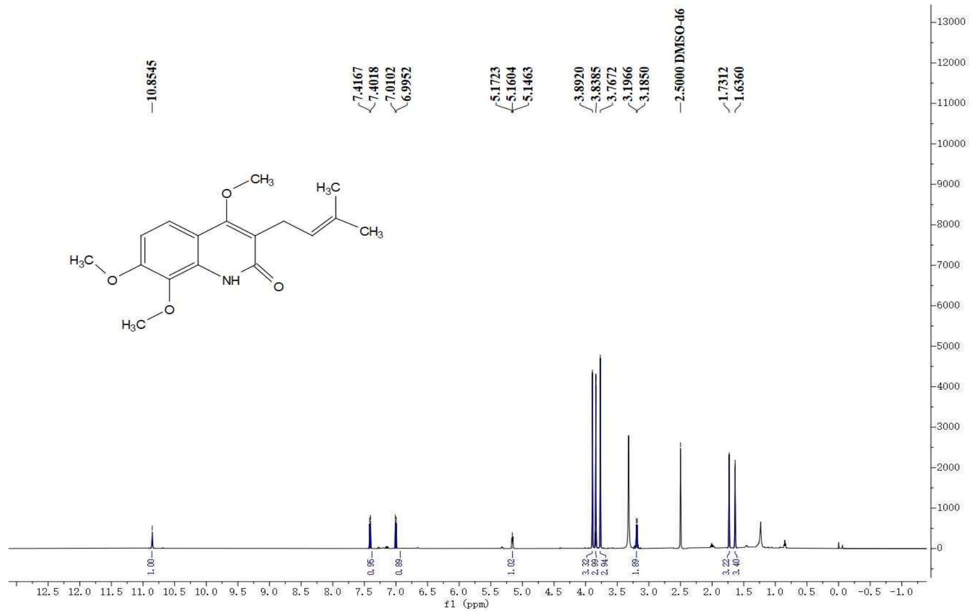

Figure S15. Compound VIII ^{1}H NMR spectrum.

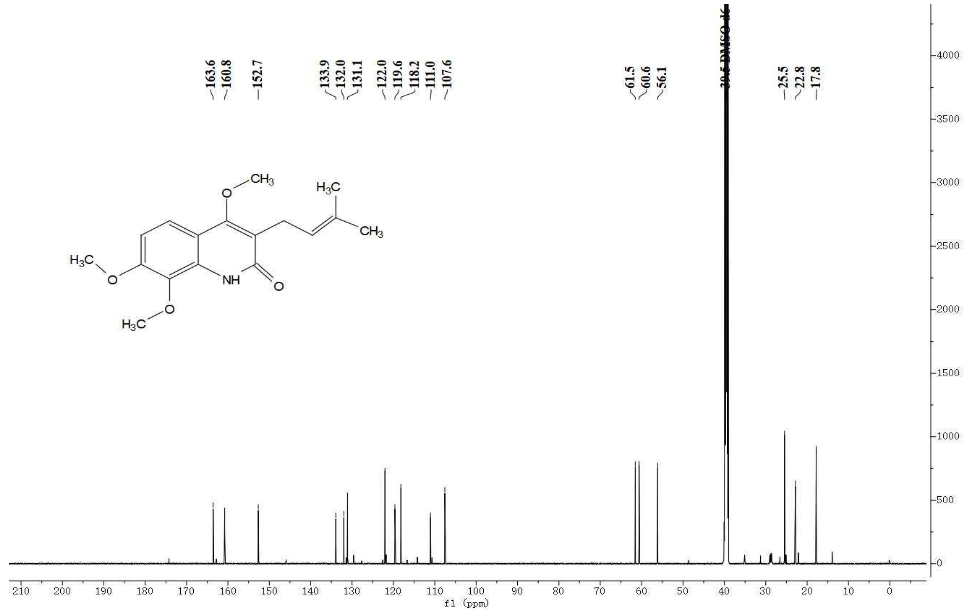

Figure S16. Compound VIII ^{13}C NMR spectrum.

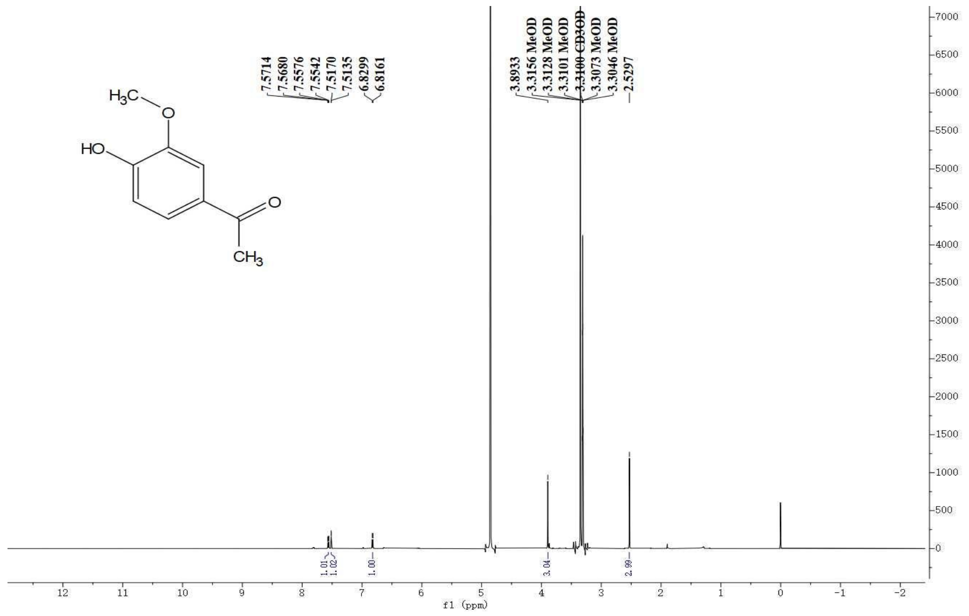

Figure S17. Compound IX ^{1}H NMR spectrum.

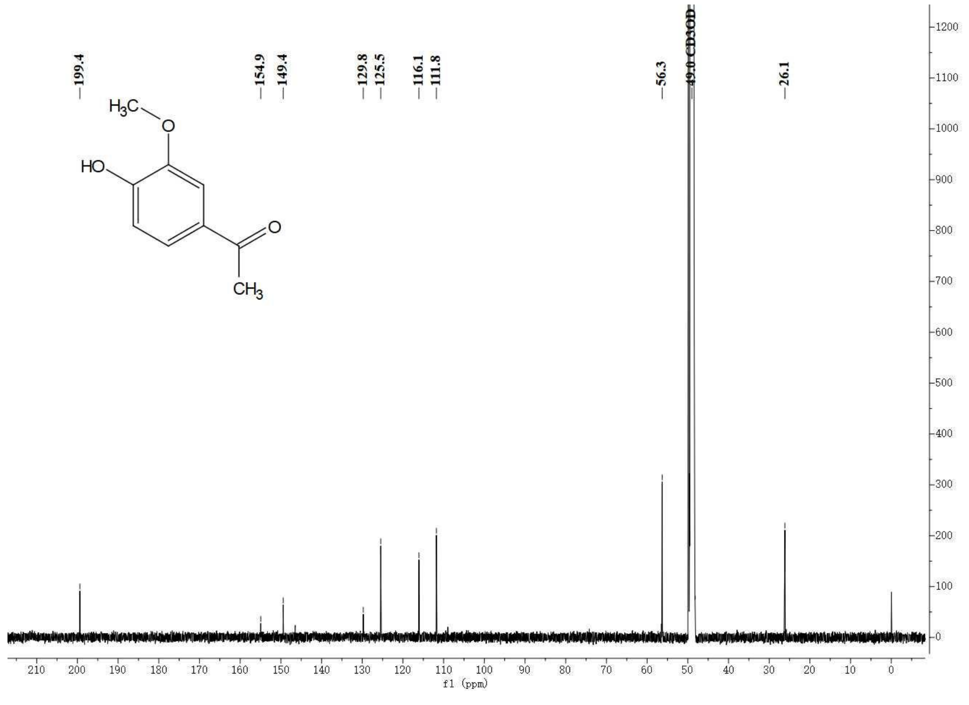

Figure S18. Compound IX ^{13}C NMR spectrum.

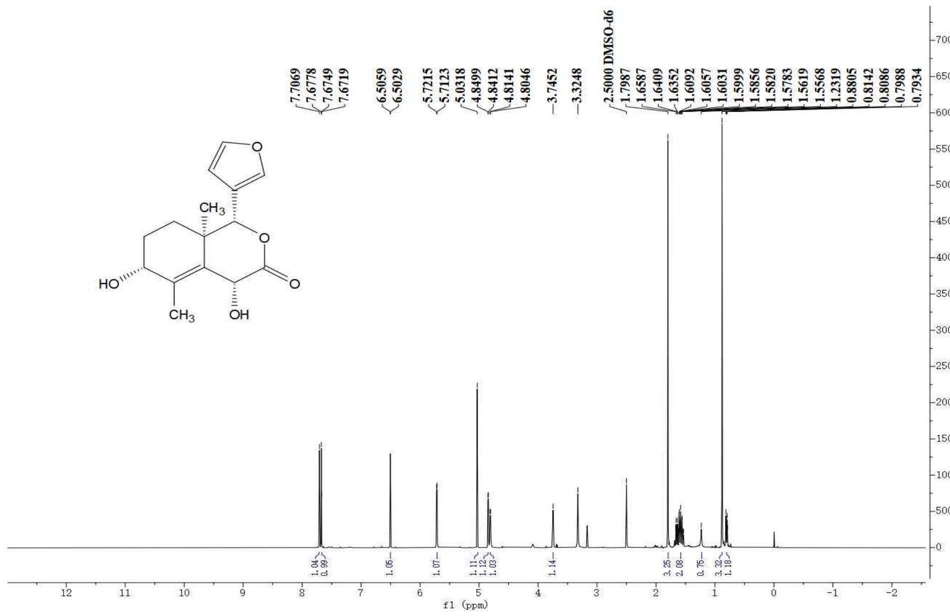

Figure S19. Compound X ^{1}H NMR spectrum.

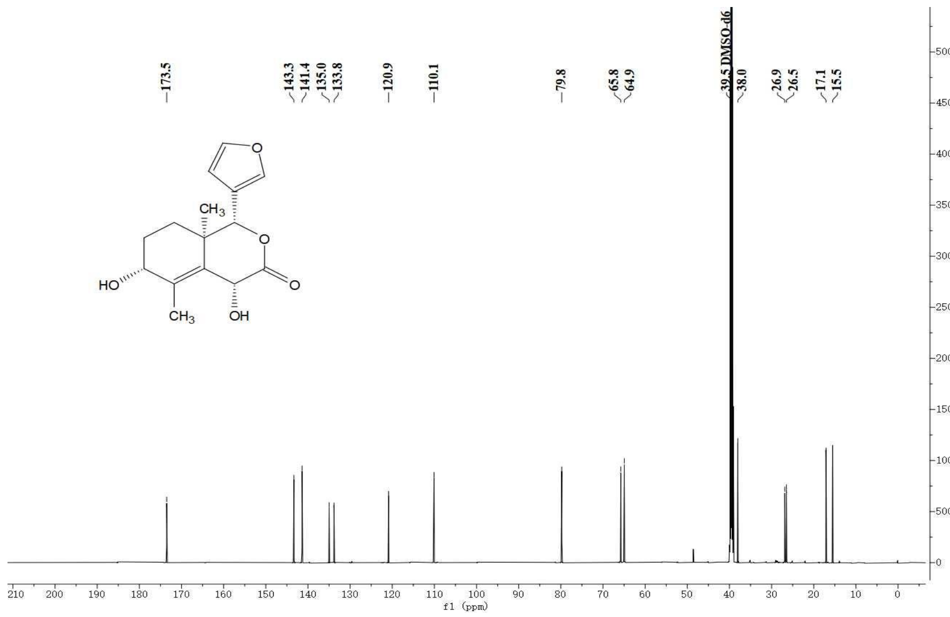

Figure S20. Compound X ^{13}C NMR spectrum.

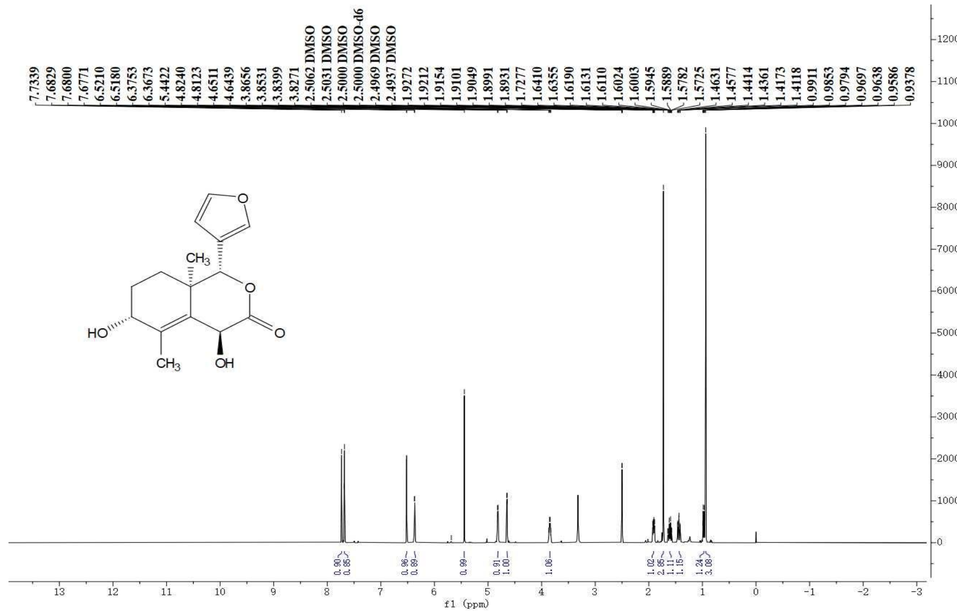

Figure S21. Compound XI ^{1}H NMR spectrum.

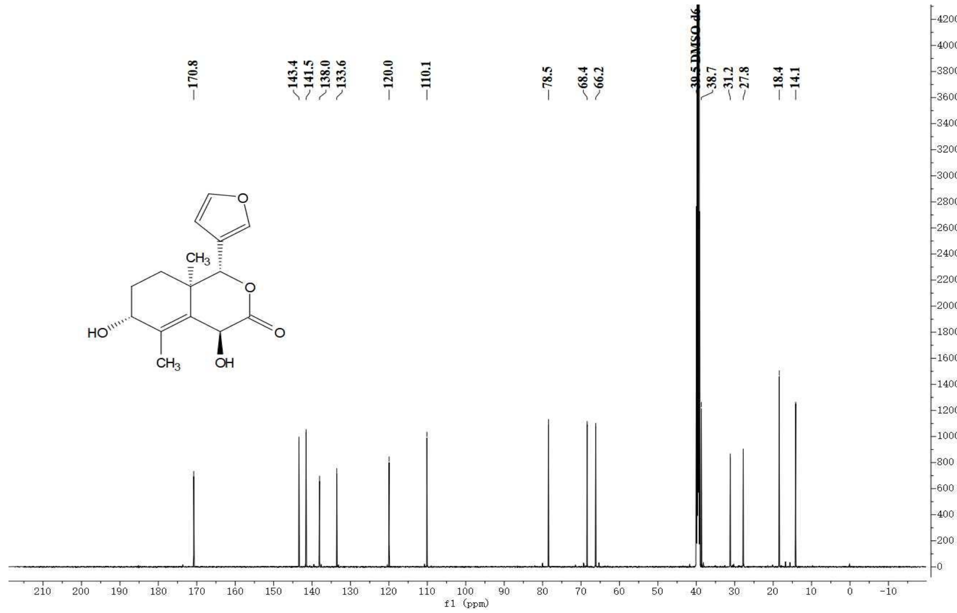

Figure S22. Compound XI ^{13}C NMR spectrum.

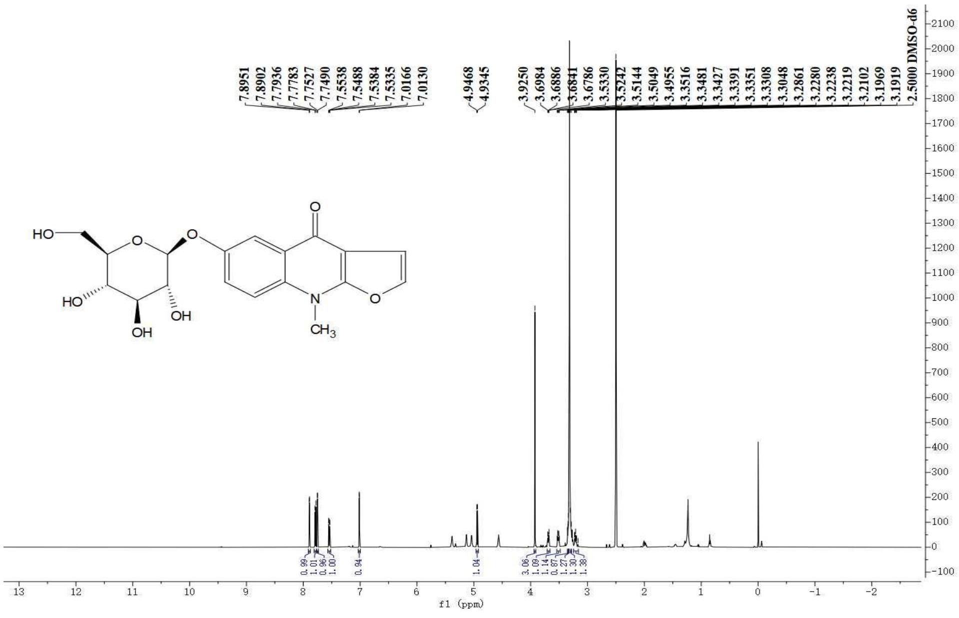

Figure S23. Compound XII ^{1}H NMR spectrum.

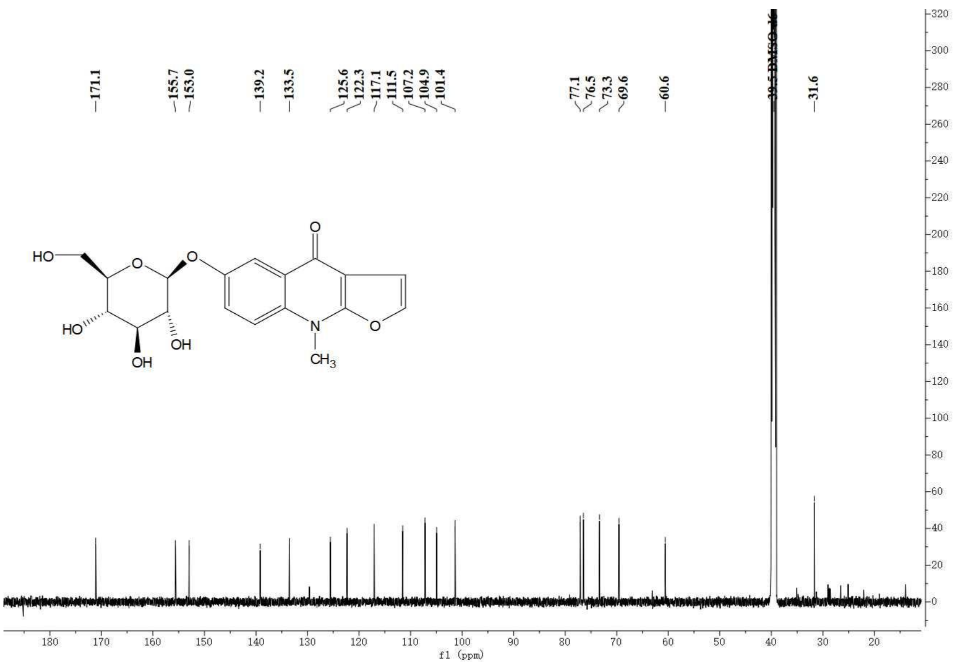

Figure S24. Compound XII ^{13}C NMR spectrum.

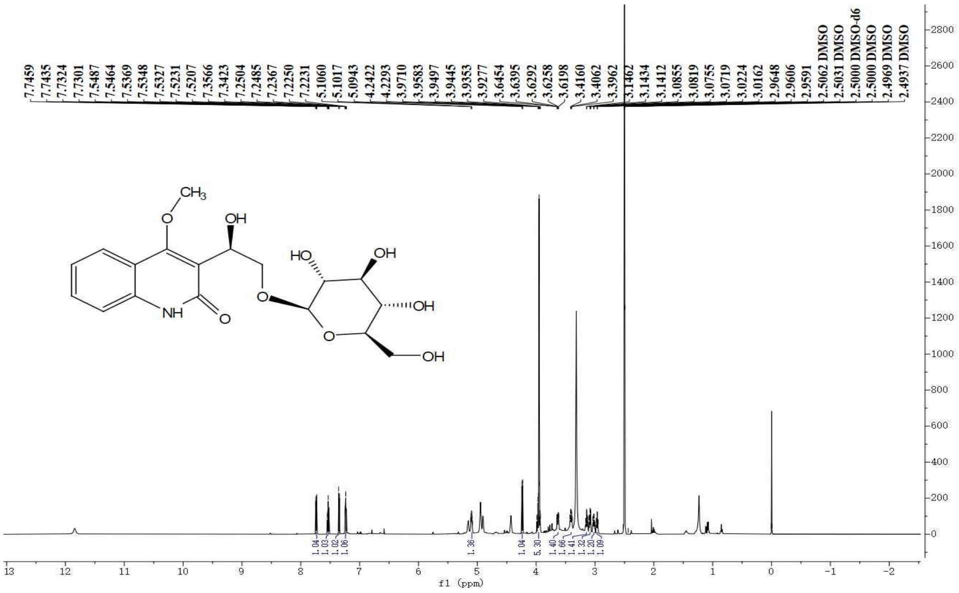

Figure S25. Compound XIII ^{1}H NMR spectrum.

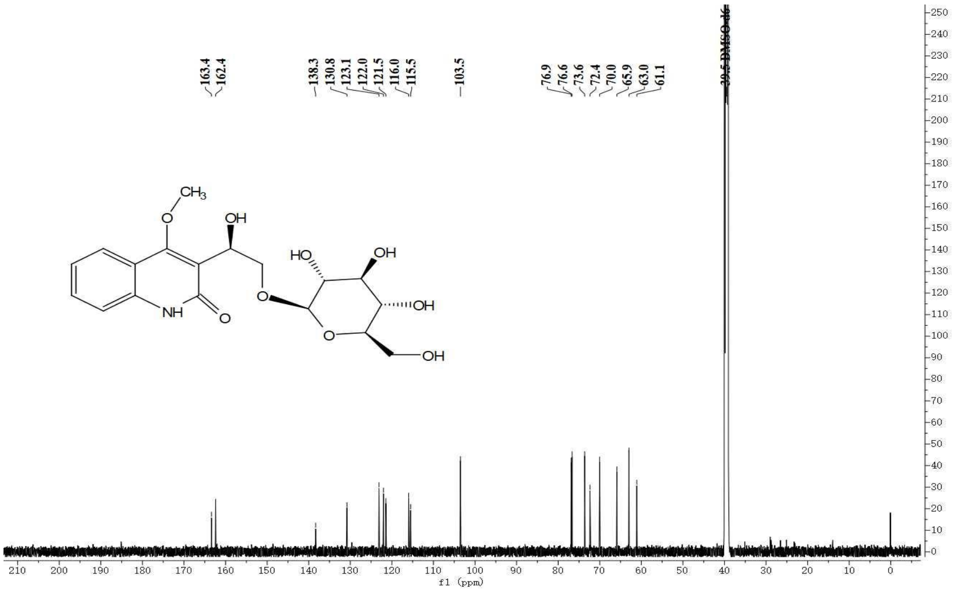

Figure S26. Compound XIII ^{13}C NMR spectrum.

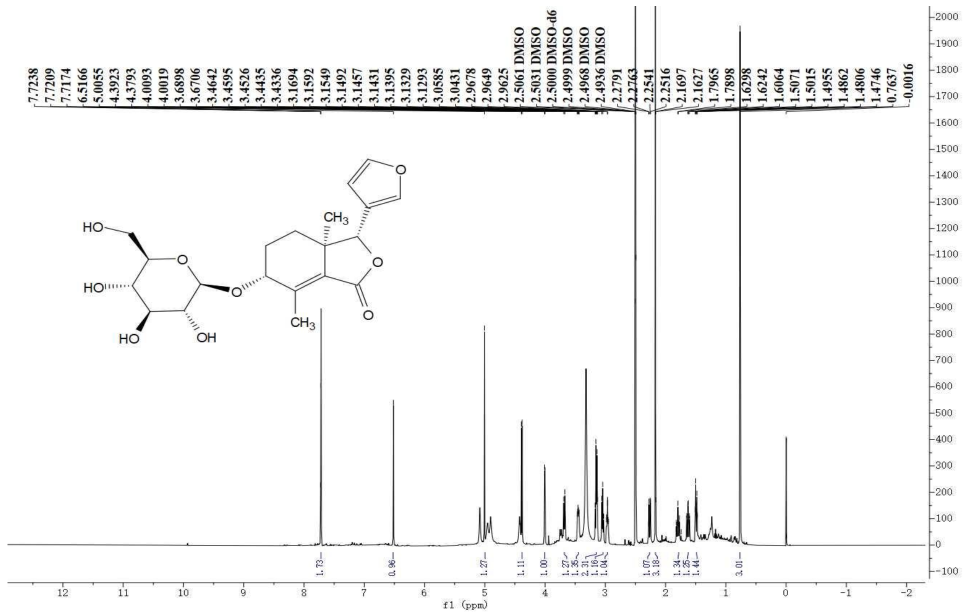

Figure S27. Compound XIV ^{1}H NMR spectrum.

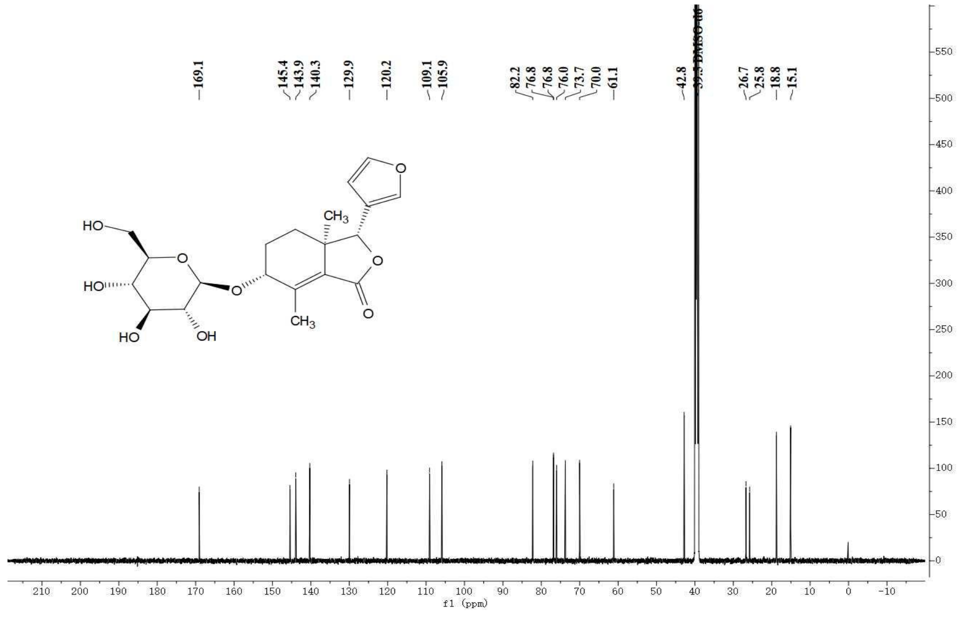

Figure S28. Compound XIV ^{13}C NMR spectrum.

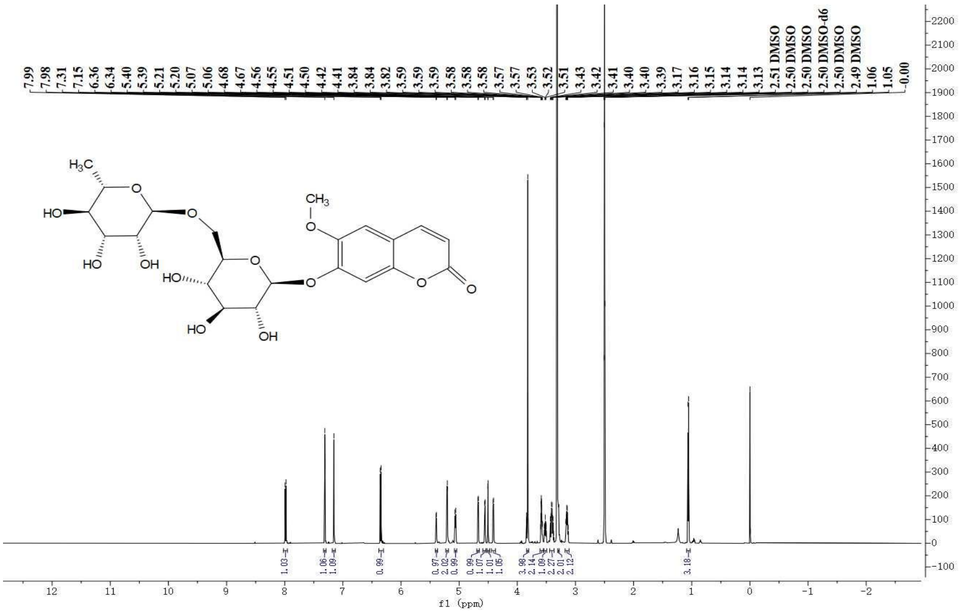

Figure S29. Compound XV ^{1}H NMR spectrum.

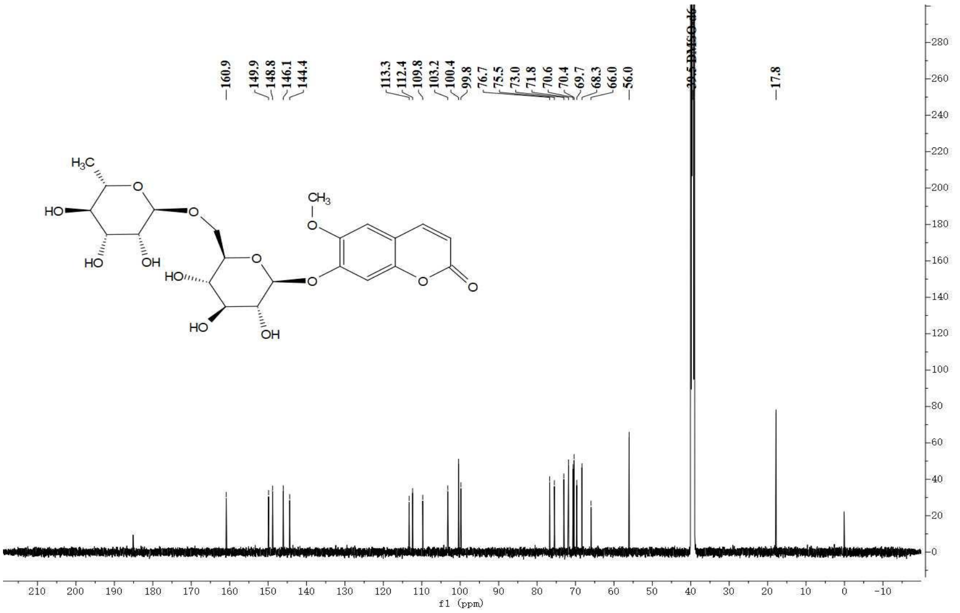

Figure S30. Compound XV ^{13}C NMR spectrum.

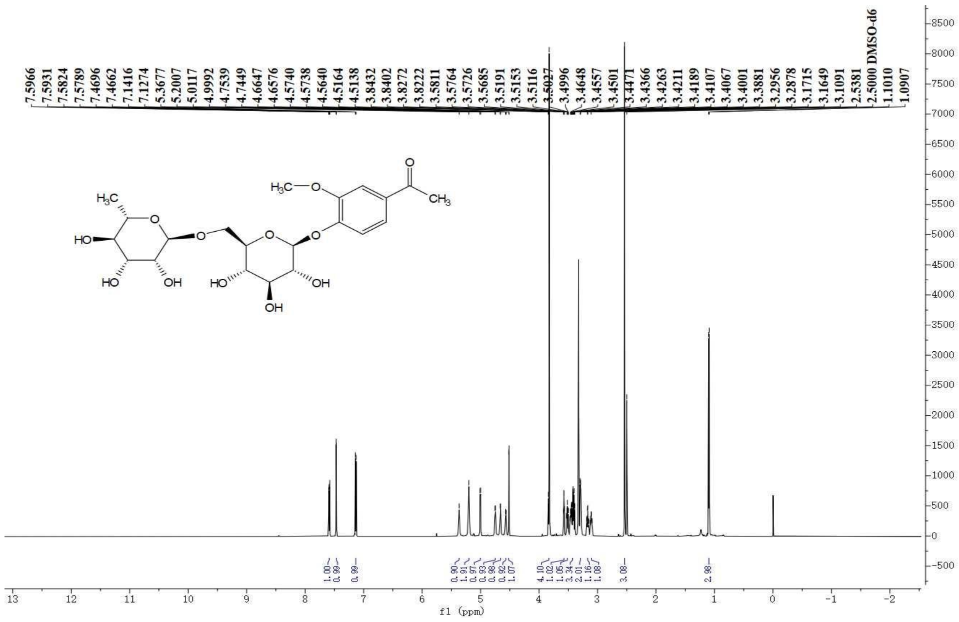

Figure S31. Compound XVI ^{1}H NMR spectrum.

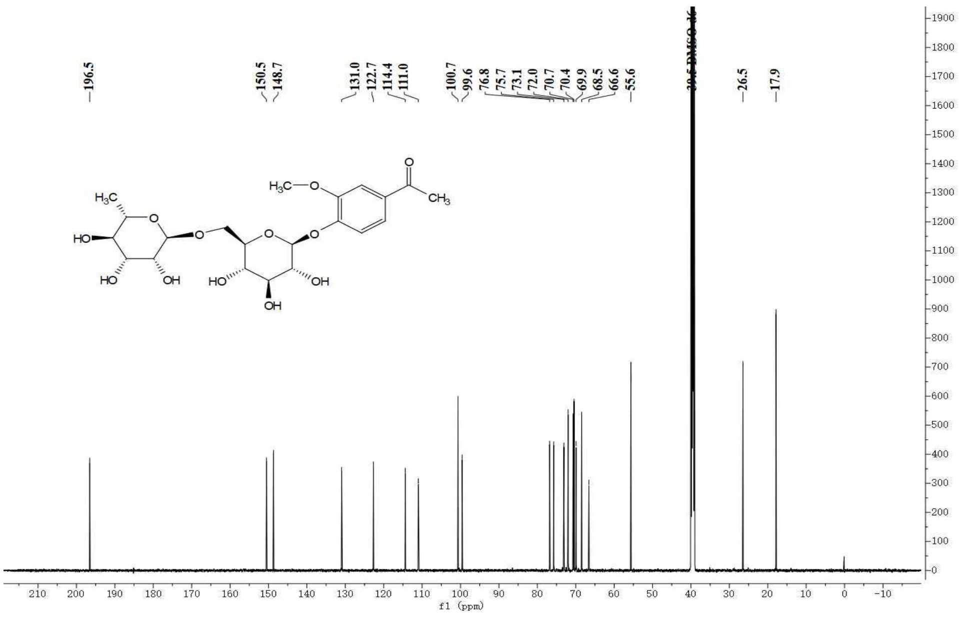

Figure S32. Compound XVI ^{13}C NMR spectrum.

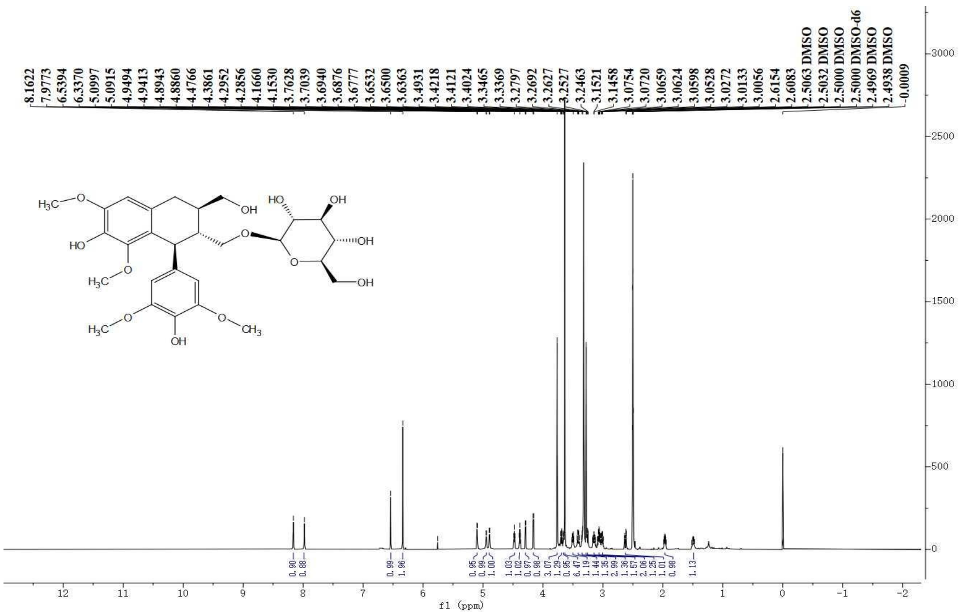

Figure S33. Compound XVII ^{1}H NMR spectrum.

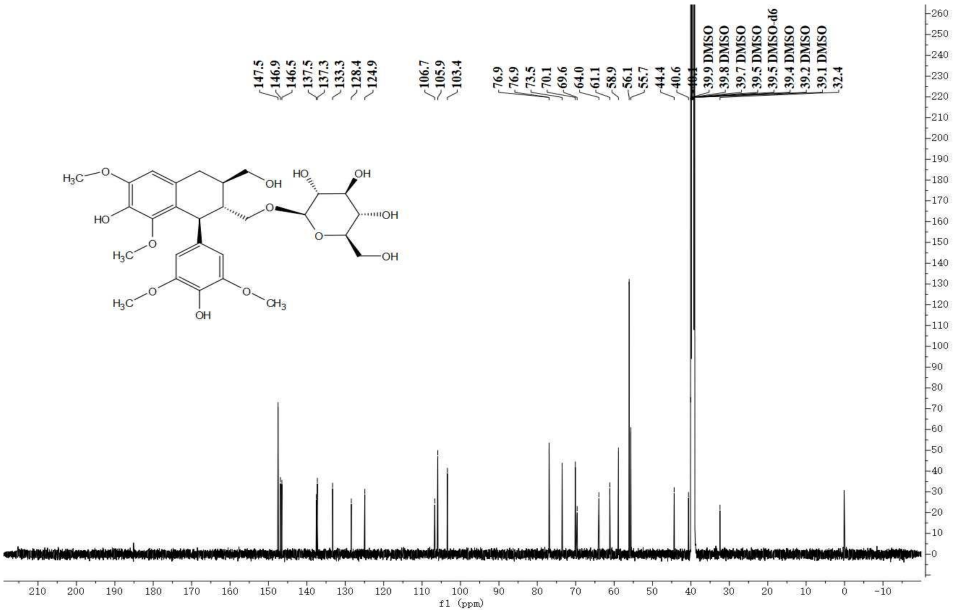

Figure S34. Compound XVII ^{13}C NMR spectrum.

Table S1. The top 25 KEGG pathways.

List	Description	Gene
hsa05417	Lipid and atherosclerosis	CASP1,MAPK14,ICAM1,JAK2,JUN,MMP9,STAT3,TLR4,TNF,TNFRSF1A,VCAM1
hsa05418	Fluid shear stress and atherosclerosis	MAPK14,ICAM1,JUN,KDR,MMP2,MMP9,TNF,TNFRSF1A,VCAM1
hsa05171	Coronavirus disease-COVID-19	CASP1,MAPK14,ACE,EGFR,JUN,STAT3,TLR4,TNF,TNFRSF1A
hsa05205	Proteoglycans in cancer	MAPK14,EGFR,KDR,MMP2,MMP9,STAT3,TLR4,TNF
hsa04933	AGE-RAGE signaling	MAPK14,ICAM1,JAK2,JUN,MMP2,STAT3,TNF,VCAM1

	pathway in diabetic complications	
hsa04668	TNF signaling pathway	MAPK14,ICAM1,JUN,MMP9,PTGS2,TNF,TNFRSF1A,VCAM1
hsa05131	Shigellosis	CASP1,MAPK14,EGFR,JUN,TLR4,TNF,TNFRSF1A
hsa05132	Salmonella infection	CASP1,MAPK14,JUN,TLR4,TNF,TNFRSF1A,TLR9
hsa05235	PD-L1 expression and PD-1 checkpoint pathway in cancer	MAPK14,EGFR,JAK2,JUN,STAT3,TLR4,TLR9
hsa05142	Chagas disease	MAPK14,ACE,JUN,TLR4,TNF,TNFRSF1A,TLR9
hsa05161	Hepatitis B	MAPK14,JAK2,JUN,MMP9,STAT3,TLR4,TNF
hsa05167	Kaposi sarcoma-associated herpesvirus infection	MAPK14,ICAM1,JAK2,JUN,PTGS2,STAT3,TNFRSF1A
hsa05163	Human cytomegalovirus infection	MAPK14,EGFR,CXCR2,PTGS2,STAT3,TNF,TNFRSF1A
hsa04217	Necroptosis	CASP1,JAK2,STAT3,TLR4,TNF,TNFRSF1A
hsa05164	Influenza A	CASP1,ICAM1,JAK2,TLR4,TNF,TNFRSF1A
hsa05140	Leishmaniasis	MAPK14,JAK2,JUN,PTGS2,TLR4,TNF
hsa05152	Tuberculosis	MAPK14,JAK2,TLR4,TNF,TNFRSF1A,TLR9
hsa05130	Pathogenic Escherichia coli infection	CASP1,MAPK14,JUN,TLR4,TNF,TNFRSF1A
hsa04064	NF-kappa B signaling pathway	ICAM1,PTGS2,TLR4,TNF,TNFRSF1A,VCAM1
hsa04620	Toll-like receptor signaling pathway	MAPK14,CTSK,JUN,TLR4,TNF,TLR9
hsa05145	Toxoplasmosis	MAPK14,JAK2,STAT3,TLR4,TNF,TNFRSF1A
hsa05144	Malaria	ICAM1,TLR4,TNF,VCAM1,TLR9
hsa05120	Epithelial cell signaling in Helicobacter pylori infection	MAPK14,EGFR,CXCR1,CXCR2,JUN
hsa05133	Pertussis	CASP1,MAPK14,JUN,TLR4,TNF
hsa04657	IL-17 signaling pathway	MAPK14,JUN,MMP9,PTGS2,TNF

Note: The pathways are sorted according to their degree values.
